# Having the Cake and Eating It Too: First-Order, Second-Order and Bifactor Representations of Work Engagement

**DOI:** 10.3389/fpsyg.2021.615581

**Published:** 2021-07-22

**Authors:** Janos Salamon, István Tóth-Király, Beáta Bõthe, Tamás Nagy, Gábor Orosz

**Affiliations:** ^1^Doctoral School of Psychology, ELTE Eötvös Loránd University, Budapest, Hungary; ^2^Institute of Psychology, ELTE Eötvös Loránd University, Budapest, Hungary; ^3^Department of Ergonomics and Psychology, Budapest University of Technology and Economics, Budapest, Hungary; ^4^Department of Psychology, Concordia University, Montreal, QC, Canada; ^5^Département de Psychologie, Université de Montréal, Montreal, QC, Canada; ^6^ULR 7369 -URePSSS - Unité de Recherche Pluridisciplinaire Sport Santé Société, Sherpas, Univ. Lille, Univ. Artois, Univ. Littoral Côte d’Opale, Lille, France

**Keywords:** work engagement, validity evidence based on test-criterion relationship, bifactor-CFA, work addiction, work satisfaction, basic psychological needs

## Abstract

Even though work engagement is a popular construct in organizational psychology, the question remains whether it is experienced as a global construct, or as its three components (vigor, dedication, absorption). The present study thus contributes to the ongoing scientific debate about the dimensionality of work engagement systematically compared one-factor, first-order, higher-order, and bifactor confirmatory factor analytic (CFA) representations of work engagement measured by the short version of Utrecht Work Engagement Scale (UWES-9). We also documented the validity evidence of the most optimal representation based on its test-criterion relationship with basic psychological need fulfillment at work, turnover intentions, work addiction, and work satisfaction. Based on responses provided by two distinct samples of employees (*N*_1_ = 242, *N*_2_ = 505), our results supported the superiority of the bifactor-CFA representation including a global factor of work engagement and three co-existing specific factors of vigor, dedication, and absorption. This representation replicated well across the two samples through tests of measurement invariance. Finally, while global work engagement was substantially related to all correlates, the specific factors also demonstrated meaningful associations over and above the global levels of work engagement.

## Introduction

Following the changes in work conditions and technological advancements over the last decades, employees invest more and more time and energy in their work (van Beek et al., 2012). This heavy work investment can be conceptualized in the form of work engagement which has been described as a positive and fulfilling, work-related state of mind (Schaufeli et al., 2002) characterized by three components: vigor (i.e., having high levels of energy during work), dedication (i.e., perceiving work as being important and meaningful), and absorption (i.e., being immersed in work). Work engagement is thus a high activation state of mind that is associated with pleasant work-related emotions (Bakker and Oerlemans, 2011). Research has generally demonstrated that work engagement is a desirable state of mind that is positively associated with psychological health (Simbula et al., 2013; Gillet et al., 2019), psychological capital (Mills et al., 2012), occupational self-efficacy (Simbula et al., 2013; Villotti et al., 2014), passion at work (Tóth-Király et al., 2021), work performance (Gorgievski et al., 2010; Alessandri et al., 2015), personal development (Simbula et al., 2013), organizational commitment (Hallberg and Schaufeli, 2006), and job satisfaction (Wefald et al., 2012; Schaufeli et al., 2019).

Despite these findings, the dimensionality of work engagement remains questionable and is frequently investigated in the scientific literature, with two perspectives being prevalent. The first perspective (e.g., Balducci et al., 2010) proposes that the three specific components of work engagement are experienced separately, while the second perspective (e.g., Alessandri et al., 2015) proposes that work engagement is often experienced holistically, as a global construct. The present study was designed with the aim of bringing together these two diverging perspectives by showing that one can “have the cake and eat it too”; that is, one could simultaneously take into account the global and specific nature of work engagement. To achieve this goal, we first compared alternative first-order, second-order, and bifactor confirmatory factor analytic (CFA) models of the 9-item Utrecht Work Engagement Scale (UWES-9; Schaufeli et al., 2006) across two distinct samples of Hungarian^1^ employees to identify the most adequate representation of work engagement. Second, via tests of measurement invariance, we investigated the generalizability of the most optimal representation across the two samples. Third, we investigated the relations between this improved representation and key work-related correlates of work engagement, namely basic psychological need fulfillment at work, turnover intentions, work addiction, and work satisfaction.

### The Dimensionality of Work Engagement

While the 17-item Utrecht Work Engagement Scale (UWES-17) was developed first by Schaufeli et al. (2002) as a measure of work engagement, the present study focuses on the shorter, 9-item version (UWES-9, Schaufeli et al., 2006) whose factor structure was investigated in numerous studies and validated in many countries. We were able to identify a total of 33 independent studies that investigated the factor structure and reliability of the UWES-9 (more details are provided in Supplementary Table 1 in the online supplements). These studies were conducted in a large variety of nations (e.g., Netherlands, Sweden, South Korea, United States, Italy) using samples that differed not just in size, but age composition as well. Generally speaking, these studies showed that the specific components of work engagement (i.e., vigor, dedication, and absorption) had at least moderate levels of internal consistency in some studies (e.g., Chaudhary et al., 2012), but also satisfactory levels of internal consistency in most studies ranging between 0.70 and 0.92.

Although studies supported the generally adequate reliability of the UWES-9, contradictory findings have been reported about its factor structure and, in turn, the dimensionality of work engagement. Findings in most of the studies (25 out of the 33) align with the first perspective about the specific work engagement components. Consequently, these studies reported support for the three-factor model as the most optimal solution, which incorporated the three intercorrelated specific components of work engagement, but not the global work engagement construct. Based on commonly used goodness-of-fit indices (such as CFI, TLI, and RMSEA), only nine out of the 25 studies (Schaufeli et al., 2006; Nerstad et al., 2009; Seppälä et al., 2009; Breevaart et al., 2012; Fong and Ng, 2012; Yusoff et al., 2013; Panthee et al., 2014; Lathabhavan et al., 2017; Moreira-Fontán et al., 2019) reported empirical support for the three-factor solution without any model modification. It is interesting to note that ten studies (Samples 1 and 2 of Littman-Ovadia and Balducci, 2013; Ho Kim et al., 2017; Kulikowski, 2019; Sample 1 of Mills et al., 2012; Wefald et al., 2012; Villotti et al., 2014; Vazquez et al., 2015; Petroviæ et al., 2017; Zeijen et al., 2018) chose the three-factor solution as the most optimal one even though the three-factor solution in these studies failed to achieve an acceptable level of fit. In the remaining six studies, the authors opted to modify the three-factor solution by including correlated uniquenesses between a subset of items (Samples 1 and 2 of Balducci et al., 2010; Chaudhary et al., 2012; Simbula et al., 2013; Zecca et al., 2015; Lovakov et al., 2017). However, the *ad hoc* inclusion of correlated uniquenesses for the artificial improvement of model fit is considered to be problematic without any substantive interpretation of why the uniquenesses of a particular subset of items should be allowed to correlate (Marsh, 2007; Marsh et al., 2010).

Despite studies supporting the relative adequacy of the three-factor solution, it has to be noted that the average correlation between vigor, dedication, and absorption was often so high (ranging from 0.57 to 0.97) that it questions the validity evidence based on relations to other variables, specifically discriminant evidence of these components. Consequently, it has been suggested in the literature that the global construct of work engagement, and not its specific components, should be in the focus of investigations. The presence of a global work engagement factor could be investigated in different ways, with the first being the estimation of a one-factor solution that only incorporates a single work engagement factor. Three studies reported this solution as the most optimal model. However, model fit indices were not unanimously adequate in these studies (study 2 of Mills et al., 2012; Vallières et al., 2017). Although the one-factor solution reported by Klassen et al. (2012) was adequate, the inclusion of correlated uniquenesses limits the adequacy of their findings. The fourth study that supported the one-factor solution (Hallberg and Schaufeli, 2006) simultaneously accepted the three-factor solution, while neither model reached an acceptable level of RMSEA.

As a second way of testing the presence of a global construct, Sinval et al. (2018) estimated a second-order model in which a global work engagement factor was responsible for the associations between the three first-order specific factors. However, the fit indices were marginally acceptable only in one of their samples, and not unanimously acceptable in another sample, suggesting that this particular representation might not be the most optimal.

Psychometrically, however, second-order models have one important limitation: they assume that the ratio of variance explained by the global factor relative to that explained by the specific factors is the same for all items related to the specific first-order factor (Reise, 2012; Gignac, 2016). This proportionality constraint, however, has been shown to be overly strict and rarely verified in practice (Gignac, 2016; Morin et al., 2016a). Alternatively, bifactor modeling has been proposed as flexible alternative that does not rely on such an unrealistic assumption. More importantly, bifactor modeling makes it possible to directly test the simultaneous presence of a global (G-) factor (i.e., global levels of work engagement underlying responses to all items) and co-existing specific (S-) factors (i.e., unique specificities not explained by the global factor).

To the best of our knowledge, there has only been a single study that tested the adequacy of bifactor solutions. de Bruin and Henn (2013) compared first-order and bifactor solutions and reported a partial bifactor solution (including 1 G- and 2 S-factors) as the most optimal. This partial bifactor model was characterized by a well-defined work engagement G-factor and two more weakly defined vigor and absorption S-factors. The authors did not estimate a third S-factor and argued that all the variance in the dedication items was absorbed by the G-factor, leaving no residual specificity to the dedication S-factor. Other studies relying on the longer version of the UWES also showed the added value of estimating a bifactor representation of work engagement (e.g., Gillet et al., 2018, 2019).

Based on these contradictory findings, there is still a debate on whether work engagement should be measured as a single overarching construct or via its three components. Bifactor modeling appears to be a promising avenue that could bring together the two diverging perspectives and show that work engagement might be characterized by a global dimension *and* co-existing specific components not explained by the global factor. The directly related findings of de Bruin and Henn (2013) and the indirectly related findings of Gillet et al. (2018, 2019) appear to lend support for our proposition, and allow us to propose the following hypothesis:

**Hypothesis 1.** The bifactor representation of work engagement will be the most optimal compared to the alternative first-order and second-order representation and it will replicate well across the two independent samples.

### Validity of Work Engagement Based on Its Test-Criterion Relationship

Beyond the structural analysis of work engagement, we also aimed to investigate its validity evidence based on test-criterion relationship (American Educational Research Association et al., 2014). For this purpose, we relied on a diverse set of theoretically relevant work-related constructs that showed meaningful associations with work engagement in prior studies, namely basic psychological need fulfillment at work, turnover intentions, work addiction, and work satisfaction.

Self-determination theory (SDT; Ryan and Deci, 2017), a macro-theory of human motivation, posits that there exist three *basic psychological needs* whose fulfillment is essential for optimal functioning, growth, and health (Deci and Ryan, 2000). The three needs are the need for autonomy (i.e., the experience of personal volition), the need for competence (i.e., the experience of mastery and efficacy), and the need for relatedness (i.e., the experience of having meaningful relationships with others). These needs are also thought to be universal, a proposition that is supported by studies conducted in the field of, for instance, education (Cox and Williams, 2008), health (Tóth-Király et al., 2019c) or sports (Adie et al., 2008). Not surprisingly, the importance of need fulfillment has also been highlighted in the domain of work (for a review, see Van den Broeck et al., 2016). There have been some studies which focused on the associations between work engagement and need fulfillment at work with most studies reporting moderate-to-strong associations between them regardless of relying on global levels of work engagement or its specific components (Shuck et al., 2015; Trépanier et al., 2015; Wang et al., 2018). The same associations remained present when reported between work engagement and basic psychological need fulfillment specific factors (Gillet et al., 2015; Goodboy et al., 2017). However, to the best of our knowledge, there are no prior studies that assessed the relationship between work engagement and need fulfillment while, at the same time, taking into account both their global and specific components.

*Turnover intentions* have long been regarded as a key variable of interest in organizations given that frequent turnovers imply substantial organizational costs both directly (e.g., constant recruitment and replacement of staff) and indirectly (e.g., the loss of organizational knowledge and the decrease in productivity; Fernet et al., 2017). Studies so far (Mills et al., 2012; Wefald et al., 2012; Lovakov et al., 2017) have reported moderate and negative associations between global levels of work engagement and turnover intentions, typically varying between −0.43 and −0.48. Albeit slightly weaker, the same associations have also been reported when studies focused on the three components of vigor (varying between −0.38 to −0.46), dedication (varying between −0.38 and −0.51), and absorption (varying between −0.31 and −0.36).

As a downside of work engagement, *work addiction* has been described as an extreme and unhealthy form of work involvement (Porter, 1996) that is associated with, for instance, psychiatric difficulties (Andreassen et al., 2016) and poorer work performance (Falco et al., 2013). From an organizational perspective (e.g., Schaufeli et al., 2009), work addiction is typically defined as an uncontrollable and compulsive need for excessive work; from a clinical perspective (Griffiths, 2005), work addiction is best understood as a constellation of components of behavioral addictions. However, recent theoretical works (Andreassen et al., 2018) acknowledge that both perspectives refer to the same underlying phenomenon. The relationship between work engagement and work addiction has been extensively investigated. Most prior studies generally showed weak, positive association between work addiction and global levels of work engagement (e.g., van Beek et al., 2012; Clark et al., 2014; Schaufeli et al., 2019) with only a few exceptions which reported either weak negative or non-significant associations (Zeijen et al., 2018; Schaufeli et al., 2019). Results become more nuanced when the specific components of work engagement are investigated. More specifically, studies typically reported work addiction having meaningful associations with the absorption component of work engagement, but not with vigor and dedication (Schaufeli et al., 2008; van Beek et al., 2012; Clark et al., 2016). The association between workaholism and absorption might be attributed to the fact that both engaged workers and workaholics are immersed in their work and might find it difficult to disengage from it.

Finally, the present study also included *work satisfaction* as it is considered to be a positive component of employee’s wellbeing at work (Ryan and Deci, 2001) that is informative of employees’ functioning (e.g., Faragher et al., 2005). Research focusing on the associations between work satisfaction and global levels of work engagement has generally shown positive relations between them as well as between work satisfaction and vigor (varying between 0.41 and 0.65), dedication (varying between 0.42 and 0.73), and absorption (varying between 0.36 and 0.58) (e.g., Schaufeli et al., 2008; Simbula et al., 2013; Littman-Ovadia et al., 2014).

Overall, these previous studies allow us to propose the following hypotheses:

**Hypothesis 2.** Global levels of work engagement will be positively related to (2a) basic psychological need fulfillment at work, (2b) work addiction, (2c) work satisfaction, and (2d) negatively to turnover intentions.

#### Research Question

Given the lack of prior studies with regards to the validity evidence of work engagement based on its test-criterion relationship of the bifactor representation of work engagement, as well as the distinctness of first-order and bifactor S-factors, we leave it as an open research question whether the S-factors in the bifactor representation will demonstrate any additional associations with the correlates over and above of the G-factor.

## Methods

### Procedure and Participants

The present study was conducted in accordance with the Declaration of Helsinki and with the approval of the Institutional Review Board of Eötvös Loránd University Faculty of Education and Psychology. Participants for this study were recruited through company mailing lists as well as through social media groups. Potential participants were informed about the content of the online survey and they had to explicitly indicate their intention for participation. Sample 1 was collected in January-September 2018 and Sample 2 was collected in January-April 2019, allowing us to minimize their overlap. Although the online survey did not collect any specific information that would make the identification of the participants possible, a duplicate check was conducted based on the combinations of the collected demographic and job-related information. This procedure showed no duplicates in either of the final databases, suggesting the presence of distinct participants in both samples. In addition, only participants working at the time of the data collection were included in the study (which was ensured by asking participants explicitly to indicate whether they worked at the time they responded to the survey).

Two samples were used in the current study. Participants in both samples were employees in a wide variety of organizations and job roles across Hungary. These samples were not representative of the population of Hungarian working adults. Sample 1, recruited between January-September 2018, consisted of 242 working adults (184 females, 76%) who were aged between 18 and 73 years (*M*_Sam__p__le__1_ = 35.81, *SD*_Sample__1_ = 13.46) and worked in different organizational levels (48 blue collars: 20%, 136 white collars: 56%, 58 managers: 24%). Sample 2, recruited between February-April 2019, consisted of 505 working adults (359 female, 71%) who were aged between 20 and 71 years (*M*_Sample__2_ = 37, *SD*_Sample__2_ = 11.27), and worked in different organizational levels (75 blue collars: 15%, 287 white collars: 57%, 143 managers: 28%).

### Measures

#### Work Engagement (Both Sample 1 and 2)

The short version of the Utrecht Work Engagement Scale (UWES-9, Schaufeli et al., 2006) was used that measures the three underlying dimensions of work engagement: vigor (three items; e.g., “At my work, I feel bursting with energy”), dedication (three items; e.g., “I am enthusiastic about my job”), and absorption (three items; e.g., “I get carried away when I’m working”). See Supplementary Appendix 1 in the online supplements for the Hungarian version. Responses were provided on a seven-point Likert-scale ranging from 1 (never) to 7 (always). The UWES-9 was adapted with a standardized translation-back translation protocol proposed by Beaton et al. (2000). Cronbach alpha values for all the factors indicated good internal consistency in both samples, ranging from 0.88 (absorption) to 0.90 (dedication) in Sample 1 and from 0.85 (vigor) to 0.90 (dedication) in Sample 2.

#### Turnover Intention (Sample 1)

A three-item scale adapted from the questionnaire developed to measure high school dropout intention (Vallerand et al., 1997; Hardre and Reeve, 2003) was used to measure workers’ turnover intentions. Items were translated following the standardized translation-back translation protocol proposed by Beaton et al. (2000) and slightly modified to reflect turnover intention in the work context (e.g., “I will likely be looking for a new job soon.”). Each item was scored on a five-point Likert-scale ranging from 1 (very uncharacteristic) to 5 (very characteristic). Cronbach’s alpha in the present study was 0.93.

#### Basic Psychological Need Fulfillment (Sample 1)

The Hungarian version (Tóth-Király et al., 2018) of the 24-item Basic Psychological Need Satisfaction and Frustration Scale (BPNSFS, Chen et al., 2015) was used to measure individuals’ work-related need satisfaction and frustration. Instructions were slightly adapted to the work context (all items started with the clause “At the workplace where I work…”), while the items themselves were used without any modification. The scale measures six factors: autonomy satisfaction (four items; e.g., “I feel that my decisions reflect what I really want.”; α = 0.78), relatedness satisfaction (four items; e.g., “I feel close and connected with other people who are important to me.”; α = 0.78), competence satisfaction (four items; e.g., “I feel I can successfully complete difficult tasks.”; α = 0.70), autonomy frustration (four items; e.g., “My daily activities feel like a chain of obligations.”; α = 0.64), relatedness frustration (four items; e.g., “I feel the relationships I have are just superficial.”; α = 0.78), and competence frustration (four items; e.g., “I have serious doubts about whether I can do things well.”; α = 0.77). Respondents indicated their level of agreement using a seven-point Likert-scale ranging from 1 (strongly disagree) to 5 (strongly agree).

#### Work Addiction (Sample 2)

The seven-item Hungarian version (Orosz et al., 2016) of the Bergen Work Addiction Scale (BWAS-H, Andreassen et al., 2012) was administered to measure work addiction based on the components model of addiction (Griffiths, 2005), including salience, tolerance, withdrawal, mood modification, tolerance, and relapse (e.g., “How often during the last year have you deprioritized hobbies, leisure activities, and exercise because of your work?”). Cronbach’s alpha for this scale was satisfactory (α = 0.78). Items were rated on a five-point scale (1 = never, 5 = always).

#### Work Satisfaction (Sample 2)

A five-item scale adapted from the Satisfaction with Life Scale (Diener et al., 1985; Martos et al., 2014) was used to measure respondents’ satisfaction with their works. Following prior applications (Fouquereau and Rioux, 2002; Tóth-Király et al., 2020), items were modified to refer to work instead of life in general (e.g., “The conditions of my work are excellent”). l. This modified scale indicated good internal consistency (α = 0.87). Respondents indicated their level of agreement using a seven-point Likert-scale ranging from 1 (strongly disagree) to 7 (strongly agree).

### Statistical Analyses

**FIGURE 1:**
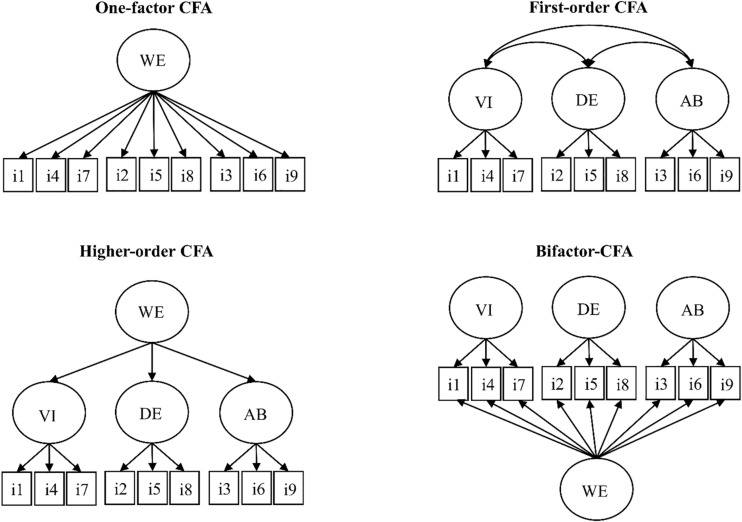
Schematic representation of the estimated model for work engagement. *Note.* CFA, confirmatory factor analysis; i1-i9, item 1-9; VI, vigor; DE, dedication; AB, absorption; WE, work engagement. Unidirectional arrows represent factor loadings, bidirectional arrows represent correlations.

Statistical analyses were performed with SPSS 22 and M*plus* 8 (Muthén and Muthén, 1998-2017). For factor analyses, the robust maximum likelihood estimator (MLR) was used as this estimator robust to non-normality and is more preferable when the response scale has more than five categories (Morin et al., 2020). The first step of the analyses comprised of the estimation of four alternative CFA solutions (see Figure 1 for a graphical depiction of these models): (1) a one-factor solution; (2) a first-order (including the 3 specific factors); (3) a second-order (including the 3 specific factors and a higher-order work engagement factor); and a (4) bifactor solution (including the 3 specific factors and a co-existing work engagement factor). All these models were estimated separately for the two samples. In the three-factor CFA solution, items were set to load only on their *a priori* specific factors, cross-loadings were set to be zero, and factors were allowed to correlate with one another. In the second-order model, specifications were the same as in the first-order model, but the correlations between the factors were replaced by a second-order global work engagement factor. In bifactor-CFA solution, items were set to load on their respective S-factors as well as on the work engagement G-factor, and following typical bifactor specifications (Reise, 2012) factors were specified as orthogonal (i.e., not allowed to correlate with one another). In the comparison of first-order and bifactor models, we followed the guidelines of Morin et al. (2016a) and apart from goodness-of-fit, we also carefully examined the standardized parameter estimates with an emphasis on the size of the correlations between the factors.

In the second stage, using the most optimal measurement model, tests of measurement invariance were conducted (Meredith, 1993; Millsap, 2011) across samples (Sample 1 vs. Sample 2) to ascertain that we relied on identical sets of indicators when investigating validity evidence based on test-criterion relationship and to test the replicability of the measurement structure. In addition, to assess the generalizability of the most optimal model to subgroups of people, we conducted the same tests of measurement invariance across groups based on gender (male vs. female), age (young adult vs. middle-old adult), and organizational level (blue collar employee vs. white collar employee vs. managers). Following typical specifications, tests of measurement invariance were conducted in a sequence where equality constraints are gradually added to the various parameters, ranging from the least restrictive model to the most restrictive one (Millsap, 2011): configural invariance (i.e., factor structure), weak invariance (i.e., factor structure and factor loadings), strong invariance (i.e., factor structure, factor loadings and intercepts), strict invariance (factor structure, factor loadings, intercepts, and uniquenesses), latent variance-covariance invariance (factor structure, factor loadings, intercepts, uniquenesses, factor variances and factor covariances), and latent mean invariance (factor structure, factor loadings, intercepts, uniquenesses, factor variances, factor covariances, and latent means).

Models were evaluated on the basis of common goodness of fit indices and interpreted along their commonly used cut-off values (Hu and Bentler, 1999; Marsh et al., 2005): the Comparative Fit Index (CFI; ≥ 0.95 good, ≥ 0.90 acceptable), the Tucker–Lewis Index (TLI; ≥ 0.95 good, ≥ 0.90 acceptable), the Root-Mean-Square Error of Approximation (RMSEA; ≤ 0.06 good, ≤ 0.08 acceptable) with its 90% confidence interval. It has to be noted the RMSEA has been shown to tends to be overinflated under conditions of low degrees of freedom (Kenny et al., 2015); therefore, this indicator is reported for the sake of transparency and comparability with previous studies, but less emphasis will be put on its interpretation. As for measurement invariance, relative changes (Δ) in the fit indices were examined (Cheung and Rensvold, 2002; Chen, 2007) where a decrease of at least 0.010 for CFI and TLI and an increase of at least 0.015 for RMSEA indicate lack of invariance. We also calculated the root deterioration per restriction (RDR; Browne and Du Toit, 1992) index which rescales the chi-square difference to approximate an RMSEA metric. Following suggestions by Raykov and Penev (1998); see also Pekrun et al., 2019), RDR was interpreted in relation to RMSEA (i.e., RDR < 0.05 indicates strong equivalence, RDR < 0.08 indicates acceptable equivalence). Spearman correlations were calculated between the factors to assess the validity evidence of the bifactor-CFA solution based on its test-criterion relationship. Reliability was assessed with the model-based omega composite reliability coefficient (McDonald, 1970; Morin et al., 2020) and values above 0.500 are considered adequate (Perreira et al., 2018). All questions were mandatory; therefore, the sample sizes were the same for all analyses. The data can be found on the following link: https://osf.io/upn9c/?view_only=8fd4125ad1654e32b7219ba29aaa0ecf.

## Results

### Structural Analysis and Measurement Invariance

Goodness-of-fit statistics of the UWES-9 can be seen in Table 1. The one-factor solution (S1M1 and S2M1) had poor fit in both samples. The three-factor CFA model (S1M2 S2M2) had marginally acceptable fit in Sample 1 (although RMSEA did not reach the minimum 0.080), and acceptable fit in Sample 2 (CFI and TLI > 0.90, RMSEA = 0.08). Correlations between the three engagement factors were high in both Sample 1 (between 0.778 and 0.887, *M* = 0.827) and Sample 2 (between 0.773 and 0.907, *M* = 0.850), suggesting conceptual redundancies between the three factors. However, the magnitude of these correlations might be inflated by an unmodeled G-factor. To test this assumption, we contrasted second-order and bifactor models (incorporating one work engagement G-factor and the three S-factors). The fit of the second-order model (S1M3 and S2M3) was identical to that of the first-order model. However, fit for the bifactor models (S1M4 and S2M4) was good (CFI and TLI > 0.95, RMSEA ≤ 0.08) and it was superior to the first-order models (Sample 1: ΔCFI = + 0.036, ΔTLI = + 0.043, ΔRMSEA = −0.036; Sample 2: ΔCFI = + 0.018; ΔTLI = + 0.021; ΔRMSEA = −0.018). The work engagement G-factor was well-defined in both samples (Sample 1: λ = 0.729 to 0.883; Sample 2: λ = 0.702 to 0.921) as were the vigor (Sample 1: λ = 0.160 to 0.602; Sample 2: λ = 0.142 to 0.513) and absorption (Sample 1: λ = 0.119 to 0.632; Sample 2: λ = 0.215 to 0.484) S-factors. In contrast, the dedication S-factor (Sample 1: λ = 0.187 to 0.399; Sample 2: λ = −0.500 to 0.042) had a comparatively weaker definition.

**TABLE 1:** Goodness-of-fit statistics of the alternative measurement models on the Hungarian version of Utrecht work engagement scale.

Model	χ^2^ (df)	CFI	TLI	RMSEA	Comparison	Δχ^2^ (df)	ΔCFI	ΔTLI	ΔRMSEA	RDR
**Sample 1**										
S1M1. One-factor CFA	215.595* (27)	0.866	0.822	0.170 [0.149,0.191]	—	—	—	—	—	—
S1M2. Three-factor CFA	102.366* (24)	0.944	0.917	0.116 [0.094,0.140]	S1M1	74.048 (3)*	+ 0.078	+0.095	−0.054	Na
S1M3. Second-order CFA	102.370* (24)	0.944	0.917	0.116 [0.094,0.140]	S1M1	74.048 (3)*	+ 0.078	+0.095	−0.054	Na
S1M4. Bifactor CFA	46.016* (18)	0.980	0.960	0.080 [0.052,0.109]	S1M2	59.795 (6)*	+ 0.036	+0.043	−0.036	Na
**Sample 2**										
S2M1. One-factor CFA	242.039* (27)	0.905	0.873	0.126 [0.111,0.140]	—	—	—	—	—	—
S2M2. Three-factor CFA	101.819* (24)	0.966	0.948	0.080 [0.064,0.096]	S2M1	111.372 (3)*	+ 0.061	+0.075	−0.046	Na
S2M3. Second-order CFA	102.537* (24)	0.965	0.948	0.080 [0.065,0.097]	S2M1	132.544 (3)*	+ 0.060	+0.075	−0.046	Na
S2M4. Bifactor CFA	53.315* (18)	0.984	0.969	0.062 [0.043,0.082]	S2M2	48.279 (6)*	+ 0.018	+0.021	−0.018	Na
**Measurement Invariance Across Gender**										
MG1. Configural invariance	84.162* (36)	0.987	0.974	0.060 [0.043,0.077]	—	—	—	—	—	—
MG2. Weak invariance	105.197* (50)	0.985	0.978	0.054 [0.040,0.069]	MG1	20.511 (14)	−0.002	+ 0.004	−0.006	0.025
MG3. Strong invariance	111.108* (55)	0.985	0.980	0.052 [0.038,0.066]	MG2	4.151 (5)	0.000	+ 0.002	−0.002	NPC
MG4. Strict invariance	117.824* (64)	0.985	0.983	0.047 [0.034,0.061]	MG3	8.382 (9)	0.000	+ 0.003	−0.005	NPC
MG5. Latent variance-covariance invariance	124.139* (68)	0.985	0.984	0.047 [0.034,0.060]	MG4	6.337 (4)	0.000	+ 0.001	0.000	0.028
MG6. Latent means invariance	131.724* (72)	0.984	0.984	0.047 [0.034,0.060]	MG5	7.675 (4)	−0.001	0.000	0.000	0.035
**Measurement Invariance Across Age**										
MA1. Configural invariance	91.675* (36)	0.985	0.969	0.064 [0.048,0.081]	—	—	—	—	—	—
MA2. Weak invariance	110.681* (50)	0.983	0.976	0.057 [0.043,0.071]	MA1	16.046 (14)	−0.002	+ 0.007	−0.007	0.014
MA3. Strong invariance	132.854* (55)	0.978	0.972	0.062 [0.048,0.075]	MA2	27.379 (5)*	−0.005	−0.004	+ 0.005	0.077
MA4. Strict invariance	155.031* (64)	0.975	0.972	0.062 [0.049,0.074]	MA3	22.213 (9)*	−0.003	0.000	0.000	0.044
MA5. Latent variance-covariance invariance	185.608* (68)	0.967	0.965	0.068 [0.056,0.080]	MA4	22.446 (4)*	−0.008	−0.007	+ 0.006	0.079
MA6. Latent means invariance	206.883* (72)	0.963	0.963	0.071 [0.060,0.082]	MA5	24.914 (4)*	−0.004	−0.002	+ 0.003	0.084
**Measurement Invariance Across Organizational Levels**										
MO1. Configural invariance^a^	116.603* (56)	0.984	0.969	0.066 [0.049,0.083]	—	—	—	—	—	—
MO2. Weak invariance^b^	144.931* (82)	0.983	0.978	0.056 [0.040,0.070]	MO1	26.965 (26)	−0.001	+ 0.009	−0.010	0.007
MO3. Strong invariance	158.536* (92)	0.982	0.979	0.054 [0.039,0.068]	MO2	12.085 (10)	−0.001	+ 0.001	−0.002	0.017
MO4. Strict invariance	184.654* (110)	0.980	0.980	0.052 [0.039,0.065]	MO3	26.692 (18)	−0.002	+ 0.001	−0.002	0.025
MO5. Latent variance-covariance invariance	232.741* (118)	0.969	0.972	0.062 [0.051,0.074]	MO4	43.116 (8)*	−0.011	−0.008	+ 0.010	0.077
MO6. Latent means invariance	269.562* (126)	0.961	0.967	0.068 [0.056,0.079]	MO5	40.437 (8)*	-0.008	−0.005	+ 0.006	0.074
**Measurement Invariance Across Samples**										
MS1. Configural invariance	154.568* (36)	0.968	0.937	0.094 [0.079,0.109]	—	—	—	—	—	—
MS2. Weak invariance	102.508* (50)	0.986	0.980	0.053 [0.038,0.068]	MS1	52.533 (14)*	+ 0.018	+0.043	−0.041	0.061
MS3. Strong invariance	107.961* (55)	0.986	0.981	0.051 [0.036,0.065]	MS2	3.305 (5)	+ 0.000	+0.001	−0.002	NPC
MS4. Strict invariance	119.706* (64)	0.985	0.983	0.048 [0.035,0.062]	MS3	12.246 (9)	−0.001	+ 0.002	−0.003	0.022
MS5. Latent variance-covariance invariance	129.531* (68)	0.984	0.983	0.049 [0.036,0.062]	MS4	9.566 (4)	−0.001	0.000	+ 0.001	0.043
MS6. Latent means invariance	138.784* (72)	0.982	0.982	0.050 [0.037,0.062]	MS5	9.496 (4)	−0.002	−0.001	+ 0.001	0.028

*p < 0.01; CFA, confirmatory factor analysis; χ^2^, Chi-square; df, degrees of freedom; CFI, comparative fit index; TLI, Tucker-Lewis Index; RMSEA, root-mean-square error of approximation; 90% CI, 90% confidence interval of the RMSEA; Δχ^2^, Robust (Satorra-Bentler) chi-square difference test (calculated from loglikelihood for greater precision); ΔCFI, change in CFI value compared to the preceding model; ΔTLI, change in the TLI value compared to the preceding model; ΔRMSEA, change in the RMSEA value compared to the pre-ceding model; RDR, root deterioration per restriction index; Na, not applicable; NPC, not possible to calculate due to the fact that the chi-square difference value is smaller than the difference in the degrees of freedom. ^a^ The residual variance of item 3 was constrained to be higher than zero in all groups to achieve identification. ^b^ The residual variance of item 3 and the variance of the dedication S-factor were constrained to be higher than zero in group 2 and 3, respectively, to achieve identification.

In the next step, measurement invariance was tested across the two samples (Models MS in Table 1) to verify the replicability of the final bifactor-CFA model (see Table 1). The configural model with no equality constraints provided a reasonably good model fit based on CFI and TLI (0.968 and 0.937, respectively), but not RMSEA (0.094). Still, the confidence interval of the latter reached the level of acceptability (i.e., 0.080), suggesting that the factor structure is reasonably similar across samples. Next, we put equality constraints on the factor loadings, which led to substantial improvements in model fit (ΔCFI = + 0.018, ΔTLI = + 0.043, ΔRMSEA = −0.041; RDR = 0.061), providing good support for the weak invariance of the bifactor-CFA measurement model. The gradual inclusion of the equality constraints on the additional parameters (i.e., intercepts, uniquenesses, latent variances and covariances, and latent means) showed that (1) CFI, TLI, and RMSEA indicated good fit on all invariance levels; (2) decreases in CFI and TLI were never above 0.010 with the highest being −0.002; (3) increases in RMSEA were never above 0.015 with the highest change being + 0.001; and (4) all RDR values remained below 0.05. Highly similar results were obtained when the bifactor-CFA was contrasted along groups based on gender (Models MG in Table 1), age (Models MA in Table 1), and organizational level (Models MO in Table 1), all of which converged on the same conclusions and thus supporting the latent mean invariance and the replicability of the bifactor-CFA solution across samples, gender, age, and organizational level.

Parameter estimates from the latent mean invariant measurement model (derived from Model MS6) are reported in Table 2. These results showed a well-defined and highly reliable work engagement G-factor (λ = 0.712 to 0.905, *M* = 0.793, ω = 0.961). Once the effect of the G-factor was taken into account, the vigor (λ = 0.144 to 0.576, *M* = 0.395, ω = 0.655) and absorption (λ = 0.156 to 0.554, *M* = 0.343, ω = 0.573) S-factors retained a meaningful amount of specificity as opposed to the dedication S-factor (λ = 0.046 to 0.465, *M* = 0.193, ω = 0.379) which retained a smaller amount of specificity. The present results suggest that the dedication items mostly reflected participants’ global levels of work engagement instead of the pure dedication associated with this S-factor over and above the G-factor. When examining a bifactor solution, it is important to keep in mind that not all S-factors should be strongly defined and that S-factors tend to be weaker in bifactor representations because the items are associated with two factors (G- and S-factors) instead of one (S-factor) as in the first-order solution. In a similar vein, it should also be kept in mind that the present model used fully latent variables (instead of manifest scale scores) which are naturally corrected for measurement error and thus the factors should be considered reliable.

**TABLE 2:** Standardized parameter estimates from the latent mean invariant bifactor-CFA solution for the Hungarian version of Utrecht work engagement scale (Model MS6).

	ENG (λ)	VIG (λ)	DED (λ)	ABS (λ)	δ
**Vigor**					
Item 1	0.745**	0.576**			0.114
Item 2	0.761**	0.465**			0.205
Item 5	0.748**	0.144**			0.419
ω		0.655			
**Dedication**					
Item 3	0.905**		0.067*		0.176
Item 4	0.884**		0.465**		0.002
Item 7	0.793**		0.046		0.369
ω			0.379		
**Absorption**					
Item 6	0.769**			0.156**	0.384
Item 8	0.712**			0.554**	0.186
Item 9	0.824**			0.319**	0.219
ω	0.961			0.573	

ENG, Work Engagement; VIG, Vigor; DED, Dedication; ABS, Absorption; CFA, Confirmatory factor analysis; λ, Factor loading; δ, Item uniqueness; ω, model-based omega composite reliability; *p < 0.05; **p < 0.01.

### Validity Evidence Based on Test-Criterion Relationship

In order to assess the validity evidence of the bifactor-CFA solution based on its test-criterion relationship, Spearman correlations were calculated between the factors. Factors were represented by factor scores (standardized with 0 mean and 1 standard deviation) derived from the latent mean invariant measurement model for work engagement and from preliminary measurement models estimated *a priori*. These preliminary measurement models also allowed us to ascertain that the correlates had adequate validity evidence and reliability (see Supplementary Appendix 2 in the online supplements for more information).

Correlations between factors of work engagement, factors of need fulfillment and turnover intention can be seen in Table 3. Global levels of work engagement positively correlated with global levels of need fulfillment (*r* = 0.561, *p* < 0.001), as well as with specific levels of autonomy satisfaction (*r* = 0.440, *p* < 0.001) and relatedness satisfaction (*r* = 0.170, *p* = 0.008), while being negatively related to specific levels of autonomy frustration (*r* = –0.249, *p* < 0.001) and turnover intentions (*r* = −0.646, *p* < 0.001). Over and above the work engagement G-factor, some of the engagement S-factors also showed additional relations with the correlates, giving support for their added value. More specifically, there was a weak positive correlation between vigor and need fulfillment G-factor (*r* = 0.178, *p* = 0.006), between dedication and autonomy satisfaction (*r* = 0.158, *p* = 0.014), and between absorption and relatedness frustration S-factors (*r* = 0.160, *p* = 0.013). In addition, the dedication S-factor negatively correlated with turnover intention (*r* = −0.150, *p* = 0.020).

**TABLE 3:** Spearman Bivariate correlations between the variables used in Sample 1 (*N* = 242).

	1	2	3	4	5	6	7	8	9	10	11
1. Work engagement G-factor	—										
2. Vigor S-factor	0	—									
3. Dedication S-factor	0	0	—								
4. Absorption S-factor	0	0	0	—							
5. Need fulfillment G-factor	0.561**	0.178**	0.052	0.095	—						
6. Autonomy satisfaction S-factor	0.440**	−0.044	0.158*	0.107	0.154*	—					
7. Relatedness satisfaction S-factor	0.170**	0.037	0.065	−0.086	0.067	0.014	—				
8. Competence satisfaction S-factor	−0.049	0.085	−0.006	0.061	0.118	−0.085	−0.042	—			
9. Autonomy frustration S-factor.	−0.249**	−0.114	0.020	0.031	−0.103	−0.009	0.095	0.127*	—		
10. Relatedness frustration S-factor	0.125	0.013	−0.008	0.160*	0.048	0.128*	0.032	0.008	−0.028	—	
11. Competence frustration S-factor	−0.091	0.030	−0.009	−0.067	−0.068	−0.024	0.056	−0.009	−0.031	−0.010	—
12. Turnover intention	−0.646**	−0.095	−0.150*	0.051	−0.569**	−0.415**	−0.219**	0.281**	0.210**	0.035	0.038

G-factor, global factor from the bifactor model; S-factor, specific factor from the bifactor model; **p < 0.01, *p < 0.05.

When taking a look on the correlations involving Sample 2 (see Table 4), there was a strong positive correlation (*r* = 0.713, *p* < 0.001) between work satisfaction and global levels of work engagement as well as a weak positive correlation between global levels of work engagement and work addiction (*r* = 0.134, *p* = 0.003). Once again, the added value of the S-factors is supported by the weak positive correlation between dedication S-factor and work satisfaction (*r* = 0.131, *p* = 0.003) and by the weak positive correlation between work addiction and absorption S-factor (*r* = 0.198, *p* < 0.001).

**TABLE 4:** Spearman Bivariate correlations between variables used in Sample 2 (*N* = 505).

	1	2	3	4	5
1. Work engagement G-factor	—				
2. Vigor S-factor	0	—			
3. Dedication S-factor	0	0	—		
4. Absorption S-factor	0	0	0	—	
5. Work addiction	0.134**	−0.045	0.071	0.198**	—
6. Work satisfaction	0.713**	0.038	0.131**	0.055	−0.035

G-factor, global factor from the bifactor model; S-factor, specific factor from the bifactor model; **p < 0.01, *p < 0.05.

## Discussion

The aim of our study was to examine the representation of work engagement (as measured by the UWES-9) and to test whether the bifactor structure of work engagement would be a more adequate and improved representation compared to alternative first-order and the second-order solutions. This approach allowed us to bridge seemingly diverging perspectives by simultaneously considering both the global and specific components of work engagement. As an additional aim, the present study also documented the validity evidence of this representation based on its test-criterion relationship with basic psychological need fulfillment at work, turnover intentions, work addiction, and work satisfaction.

### The Bifactor Representation of Work Engagement

Our results, in line with Hypothesis 1, supported the superiority of the bifactor representation of work engagement, thus also aligning with findings reported by de Bruin and Henn (2013) as well as Gillet et al. (2018, 2019). In addition, the bifactor representation was well-replicated across the two distinct samples. In this bifactor representation, the G-factor can be seen as a direct reflection of employees’ global level of work engagement, while the S-factors are posited to reflect the presence of employees’ vigor, dedication, and absorption over and above, and independently from, their global levels of engagement. These specific dimensions also reflect the extent to which vigor, dedication and absorption deviate from the global levels of engagement. Previous studies using the UWES suggested that researchers should focus on using either the global or the specific components. However, our study shows that the two approaches are not mutually exclusive. Indeed, our study illustrates why it is important to carefully compare alternative measurement models in terms of model fit and standardized parameter estimates. The first-order CFA results demonstrated similar patterns to previous studies (e.g., Wefald et al., 2012; Littman-Ovadia and Balducci, 2013; Zeijen et al., 2018; Kulikowski, 2019) in that model fit was less than optimal across the two samples. Correlations between the three first-order factors were high, suggesting the potential presence of an unmodelled G-factor. By contrast, the fit indices for the bifactor solutions, which does incorporate a work engagement G-factor, were good in both samples.

Inspection of the parameter estimates associated with the bifactor model revealed a well-defined work engagement global factor, with a meaningful amount of specificity being retained in the vigor and absorption S-factors, and a smaller amount of specificity in the dedication S-factor. The weaker representation of the specific factors in the bifactor solutions can be attributed to scale items being associated with a specific and a global factor simultaneously. The small amount of specificity of the items of the dedication factor suggests that these items mostly reflected participants’ global sense of work engagement. However, this particular result does not mean that the bifactor model is not optimal or that the dedication S-factor should be discarded. Indeed, as stated by Morin et al. (2016a), it is rare to observe that all S-factors are well-defined in bifactor solutions which typically include at least some well-defined S-factors apart from a strongly defined G-factor. A weaker S-factor shows that a subset of items only serves to reflect global levels of work engagement, and this weaker S-factor simply should be interpreted with caution. While it has been argued that partial bifactor solutions should be pursued in the case of weaker S-factors (de Bruin and Henn, 2013; Fong and Ho, 2015), we argue that the meaningfulness of the G- and S-factors should be tested in relation to theoretically-relevant correlates before removing any S-factors as these investigation might support the added value of the S-factors over and above the G-factor.

### Test-Criterion Relationship Based Validity of the Bifactor Representation

#### Global Levels of Work Engagement

Our findings with respect to the validity evidence based on test-criterion relationship of the UWES-9 do not only highlight the importance of the global levels of work engagement, but also the added value of the specific levels of vigor, dedication, and absorption. More specifically, global levels of work engagement demonstrated a positive association with global levels of need fulfillment (e.g., Trépanier et al., 2015), providing support for Hypothesis 2a. These results suggest that experiencing high global levels of work engagement tend to be positively associated with experiencing high global levels of need fulfillment at work. When employees’ basic psychological needs are fulfilled at their workplace, they are more likely to experience growth, wellness, and optimal functioning (Ryan and Deci, 2017) which can translate into functioning more effectively at work and experiencing higher levels of positive work-related states such as work engagement. Both cross-sectional (e.g., Trépanier et al., 2013) and longitudinal (e.g., Trépanier et al., 2015) studies have reported need fulfillment to be an important predictor of work engagement. Over and above the global levels of need fulfillment, global work engagement was also associated with high specific levels of autonomy satisfaction and relatedness satisfaction. Experiencing high levels of engagement at work thus might not only be related to global levels of need fulfillment, but also specific levels of autonomy and relatedness satisfaction, suggesting that engaged employees tend to experience high levels of autonomy and relatedness satisfaction over and above the global levels of work engagement.

In addition to these findings, global levels of work engagement were negatively related to specific levels of autonomy frustration and turnover intentions which is in line with previous empirical studies (e.g., Trépanier et al., 2013; Shuck et al., 2015; Wang et al., 2018) that relied on first-order representations of work engagement. These results highlight that the frustrated need for autonomy (i.e., feelings of pressure and conflict at work) might have a negative effect on employees’ work engagement. Such need frustrated experiences might be attributed to need thwarting work conditions (Vansteenkiste and Ryan, 2013) in which employees are expected to behave in a certain way and have less control over what and how they need to do in their work, thus they cannot act in a volitional manner. Prior studies have already provided support for this explanation (e.g., Deci et al., 2001; Van den Berghe et al., 2016; see Deci et al., 2017 for an overview). Finally, the negative association between global levels of work engagement and turnover intentions is consistent with Hypothesis 2d, and is also in line with results of prior studies (e.g., Mills et al., 2012; Wefald et al., 2012; Lovakov et al., 2017). Thus, when employees do not feel engaged in their work, they might be more likely to detach themselves from the organization and potentially leave it.

Global levels of work engagement showed a positive and weak association with work addiction which is in line with Hypothesis 2b. This result is consistent with the results reported in most previous studies (e.g., van Beek et al., 2012; Clark et al., 2014; Littman-Ovadia et al., 2014; Di Stefano and Gaudiino, 2018). Even though this association was positive, its magnitude remained small which further supports the idea that global levels of work engagement and work addiction reflect two distinct construct that are relatively independent from one another. Additionally, global work engagement also showed a positive association with work satisfaction (i.e., engaged employees were more likely to be satisfied with their work), thus providing empirical support for Hypothesis 2c and further establishing the validity evidence of this representation. This result also corroborates findings reported in cross-sectional (e.g., Klassen et al., 2012; Littman-Ovadia and Balducci, 2013; Schaufeli et al., 2019) and meta-analytic (Christian et al., 2011) studies. While these constructs share conceptual similarities (i.e., the value of pleasure at work), they differ from one another in two main characteristics. First, they differ in their level of activation: work engagement is characterized by high level of energy as opposed to the low energy level in work satisfaction (Bakker and Oerlemans, 2011). Second, they have different sources of origin: work engagement is an affective outcome of work experience, while work satisfaction is an attitude toward work, which is based on the evaluation of conditions and characteristics of work (Christian et al., 2011; Salanova et al., 2014; Schaufeli et al., 2019).

#### Specific Levels of Work Engagement

Finally, our results also answered our Research Question by showing that some of the specific components of work engagement appeared to have an added value by demonstrating meaningful associations with the correlates. First, specific levels of *vigor* were positively related to global levels of need fulfillment at work. This result suggests that employees experiencing fulfilled basic psychological needs at work might have more work-related energy and mental resilience beyond the global levels of work engagement. Second, specific levels of *dedication* were positively related to specific levels of autonomy satisfaction and work satisfaction, but negatively to turnover intentions. These relationships suggest that by perceiving work as significant, inspiring, and meaningful (over and above the global levels of work engagement) might stem from having ample amount of choice and self-initiation at work, and it could also be protective of negative outcomes (i.e., lower levels of turnover intentions) and conductive of positive outcomes (i.e., higher levels of work satisfaction). Third, specific levels of *absorption* were positively related to specific levels of relatedness frustration. That is, when employees experience social rejection and exclusion at work by coworkers or supervisors, they might be more likely to become immersed in and obsessed with their work. This finding is consistent with prior studies (e.g., Tóth-Király et al., 2019b) documenting the potentially negative effects associated with relatedness frustration. This result is less surprising when we take into account that being isolated and lonely have already been related to decreased wellbeing and other maladaptive outcomes (e.g., Mellor et al., 2008; Kim et al., 2009). Becoming over-engaged with work (i.e., having high specific levels of absorption) might become a compensatory behavior for employees in order to counter the experiences of need frustration (Vansteenkiste and Ryan, 2013; Tóth-Király et al., 2019a; Bõthe et al., 2020). Specific levels of absorption, similar to prior findings relying on first-order factors (Líbano et al., 2012; Shimazu et al., 2015; Clark et al., 2016; Di Stefano and Gaudiino, 2018), were also positively related to work addiction. This positive relationship highlights the shared nature of absorption and work addiction as both are characterized with an immersion into the work-related activities from which it is difficult to disengage.

Overall, the present two-study investigation shows that work engagement might be best represented by a bifactor solution incorporating an overarching work engagement construct underlying all responses, as well as the three components of vigor, dedication, and absorption. Failure to taking into account this representation might lead to erroneous conclusions due to the high associations (i.e., multicollinearity) between the three work engagement components that appear to reflect a more global construct, while also masking the potential complementary effect of the S-factors beyond the G-factor. For these reasons, we would advise researchers to, in their pursuits, consider relying on fully latent measurement models that do not only make it possible to estimate the most optimal bifactor representation of work engagement, but they are also naturally corrected for measurement error. When the sample size is modest, similar to our approach, researchers could rely on factor scores derived from the bifactor measurement model in order to preserve its underlying nature (Morin et al., 2016b). In practical terms, this approach allows researchers to obtain a more precise and direct estimate of global work engagement as bifactor models weight items based on their contribution to the factor itself. To make this process seamless, as suggested by Perreira et al. (2018), automated scoring procedures could be developed, or the Mplus statistical package could be used, which has the advantage of providing standardized measurements interpretable as a function of the sample mean and standard deviation.

### Strengths and Limitations

The current study provides an alternative solution to the debate about the appropriate representation of work engagement. While the bifactor-CFA solution was the most optimal in comparison to other alternative models, it also allows us to investigate the nature of work engagement both on the global and the specific level. An additional strength is the replication of our findings using an independent second sample. The current study also documented the validity evidence of bifactor-CFA representation of work engagement based on its test-criterion relationship which was an important step toward its better understanding.

Nevertheless, there are some limitations that should be considered. Both studies were cross-sectional, implying that causality cannot be inferred from our results. Given that self-reported measures were used, responses might have been biased (e.g., social desirability). Future longitudinal research would be necessary to give a deeper understanding of how the representation of work engagement changes over time. Alternatively, it would be important to complement the present results with longitudinal or intervention studies with enhanced methodological quality (Chacón-Moscoso et al., 2016). The generalization of the current results requires their replication on a larger, international sample. Moreover, the sample consisted of mostly female and white-collar/manager participants; therefore, the sample is not representative of the Hungarian population. Future studies should verify the findings on a representative and more diverse sample (e.g., a sample including health care professionals and respondents from other occupations). Further studies focusing on examining the bifactor-CFA representation should be conducted in other countries and languages as well. Future studies would also do well in re-assessing the validity evidence based on test-criterion relationship using different work-related measures. It would also be interesting to examine the representation of engagement towards other activities such as studies (Dierendonck et al., 2021) or job (Gillet et al., 2020). Given that the dedication S-factor had relatively low reliability, future studies should investigate whether this is a re-occurring phenomenon or whether it is a sample-specific result.

## Conclusion

Taken together, the present research demonstrated the superiority of the bifactor solution, which not only provides an improved representation of work engagement, but also a clearer picture of the different relations of the global and specific components of work engagement to other, relevant work-related constructs. The importance of the specific factors of work engagement were illustrated by their diverse relations with these correlates. The results supported the discriminant validity evidence of vigor, dedication, and absorption as specific factors. The current findings support the simultaneous application of the global work engagement construct and its specific components.

## Data Availability Statement

The data that support the findings of this study are openly available in OSF at https://osf.io/upn9c/?view_only=2c0e7f703d2942438a630e7807f877b3.

## Ethics Statement

The current study was reviewed and approved by the Institutional Review Board of Eötvös Loránd University Faculty of Education and Psychology. The patients/participants provided their written informed consent to participate in this study. Informed consent was obtained from all participants included in the study.

## Author Contributions

JS and IT-K contributed to the study design, literature review, data gathering, manuscript writing, and to the data analyses and interpretation. BB, GO, and TN contributed to the literature review, and to the manuscript writing. All authors commented on the draft and contributed to the final version, approved the publication of the manuscript, and agreed to be accountable for all aspects of the work.

## Conflict of Interest

The authors declare that the research was conducted in the absence of any commercial or financial relationships that could be construed as a potential conflict of interest.

## Footnotes

1 We carried out this study in Hungary which provided us with a unique context for multiple reasons. First, recent national surveys show that Hungarian people spend a lot of time with work, around 43-44 hours per week (Kun et al., 2020; Urbán et al., 2019). Second, at the same time, Hungarian employees are substantially less engaged with their work when compared to other European countries (Schaufeli, 2018). This discrepancy (i.e., working a lot but not being engaged with it) thus creates a unique research environment that could provide further insights into the nature of work engagement.

## References

[B1] Adie J. W. Duda J. L. Ntoumanis N. (2008). Autonomy support, basic need satisfaction and the optimal functioning of adult male and female sport participants: a test of basic needs theory. *Motiv. Emot.* 32 189–199. 10.1007/s11031-008-9095-z

[B2] Alessandri G. Borgogni L. Schaufeli W. B. Caprara G. V. Consiglio C. (2015). From positive orientation to job performance: the role of work engagement and self-efficacy beliefs. *J. Happiness Stud.* 16 767–788. 10.1007/s10902-014-9533-4

[B3] American Educational Research Association, American Psychological Association, and National Council on Measurement in Education (2014). *Standards for Educational and Psychological Testing.* Washington, USA: American Educational Research Association.

[B4] Andreassen C. S. Griffiths M. D. Hetland J. Pallesen S. (2012). Development of a work addiction scale: development of a work addiction scale. *Scand. J. Psychol.* 53 265–272. 10.1111/j.1467-9450.2012.00947.x 22490005

[B5] Andreassen C. S. Griffiths M. D. Sinha R. Hetland J. Pallesen S. (2016). The relationships between workaholism and symptoms of psychiatric disorders: a large-scale cross-sectional study. *PLoS One* 11:e0152978. 10.1371/journal.pone.0152978 27192149 PMC4871532

[B6] Andreassen C. S. Schaufeli W. B. Pallesen S. (2018). Myths about “The myths about work addiction”: commentary on: ten myths about work addiction (Griffiths et al., 2018). *J. Behav. Addict.* 7 858–862. 10.1556/2006.7.2018.126 30556780 PMC6376365

[B7] Bakker A. B. Oerlemans W. G. M. (2011). “Subjective well-being in organizations,” in *Handbook of Positive Organizational Scholarship*, eds Cameron K. Spreitzer G. (Oxford: Oxford University Press).

[B8] Balducci C. Fraccaroli F. Schaufeli W. B. (2010). Psychometric properties of the Italian version of the Utrecht Work Engagement Scale (UWES-9): a cross-cultural analysis. *Eur. J. Psychol. Assess.* 26 143–149. 10.1027/1015-5759/a000020

[B9] Beaton D. E. Bombardier C. Guillemin F. Ferraz M. B. (2000). Guidelines for the process of cross-cultural adaptation of self-report measures. *Spine* 25 3186–3191. 10.1097/00007632-200012150-00014 11124735

[B10] Bõthe B. Tóth-Király I. Potenza M. N. Orosz G. Demetrovics Z. (2020). High-frequency pornography use may not always be problematic. *J. Sex. Med.* 17 793–811. 10.1016/j.jsxm.2020.01.007 32033863

[B11] Breevaart K. Bakker A. B. Demerouti E. Hetland J. (2012). The measurement of state work engagement: a multilevel factor analytic study. *Eur. J. Psychol. Assess.* 28 305–312. 10.1027/1015-5759/a000111

[B12] Browne M. W. Du Toit S. H. C. (1992). Automated fitting of nonstandard models. *Multivar. Behav. Res.* 27 269–300. 10.1207/s15327906mbr2702_13 26825725

[B13] Chacón-Moscoso S. Sanduvete-Chaves S. Sánchez-Martín M. (2016). The development of a checklist to enhance methodological quality in intervention programs. *Front. Psychol.* 7:1811. 10.3389/fpsyg.2016.01811 27917143 PMC5114299

[B14] Chaudhary R. Rangnekar S. Barua M. K. (2012). Psychometric evaluation of Utrecht work engagement scale in an Indian sample. *Asia-Pac. J. Manag. Res. Innov.* 8 343–350. 10.1177/2319510X1200800314

[B15] Chen B. Vansteenkiste M. Beyers W. Boone L. Deci E. L. Van der Kaap-Deeder J. (2015). Basic psychological need satisfaction, need frustration, and need strength across four cultures. *Motiv. Emot.* 39 216–236. 10.1007/s11031-014-9450-1

[B16] Chen F. F. (2007). Sensitivity of goodness of fit indexes to lack of measurement invariance. *Struct. Equ. Modeling* 14 464–504. 10.1080/10705510701301834

[B17] Cheung G. W. Rensvold R. B. (2002). Evaluating goodness-of-fit indexes for testing measurement invariance. *Struct. Equ. Modeling* 9 233–255.

[B18] Christian M. S. Garza A. S. Slaughter J. E. (2011). Work engagement: a quantitative review and test of its relations with task and contextual performance. *Pers. Psychol.* 64 89–136. 10.1111/j.1744-6570.2010.01203.x

[B19] Clark M. A. Michel J. S. Stevens G. W. Howell J. W. Scruggs R. S. (2014). Workaholism, work engagement and work-home outcomes: exploring the mediating role of positive and negative emotions: workaholism, work engagement and work-home. *Stress Health* 30 287–300. 10.1002/smi.2511 23913863

[B20] Clark M. A. Michel J. S. Zhdanova L. Pui S. Y. Baltes B. B. (2016). All work and no play? a meta-analytic examination of the correlates and outcomes of workaholism. *J. Manag.* 42 1836–1873. 10.1177/0149206314522301

[B21] Cox A. Williams L. (2008). The roles of perceived teacher support, motivational climate, and psychological need satisfaction in students’ physical education motivation. *J. Sport Exerc. Psychol.* 30 222–239. 10.1123/jsep.30.2.222 18490792

[B22] de Bruin G. P. Henn C. M. (2013). Dimensionality of the 9-Item Utrecht Work Engagement Scale (UWES-9). *Psychol. Rep.* 112 788–799. 10.2466/01.03.PR0.112.3.788-799 24245073

[B23] Deci E. L. Olafsen A. H. Ryan R. M. (2017). Self-determination theory in work organizations: the state of a science. *Annu. Rev. Organ. Psychol. Organ. Behav.* 4 19–43. 10.1146/annurev-orgpsych-032516-113108

[B24] Deci E. L. Ryan R. M. (2000). The “What” and “Why” of goal pursuits: human needs and the self-determination of behavior. *Psychol. Inq.* 11 227–268. 10.1207/S15327965PLI1104_01

[B25] Deci E. L. Ryan R. M. Gagné M. Leone D. R. Usunov J. Kornazheva B. P. (2001). Need satisfaction, motivation, and well-being in the work organizations of a former eastern bloc country: a cross-cultural study of self-determination. *Pers. Soc. Psychol. Bull.* 27 930–942. 10.1177/0146167201278002

[B26] Di Stefano G. Gaudiino M. (2018). Differential effects of workaholism and work engagement on the interference between life and work domains. *Eur. J. Psychol.* 14 863–879. 10.5964/ejop.v14i4.1626 30555590 PMC6266527

[B27] Diener E. Emmons R. A. Larsen R. J. Griffin S. (1985). The satisfaction with life scale. *J. Pers. Assess.* 49 71–75. 10.1207/s15327752jpa4901_13 16367493

[B28] Dierendonck C. Tóth-Király I. Morin A. J. S. Kerger S. Milmesiter P. Poncelet D. (2021). Testing associations between global and specific levels of student academic motivation and engagement in the classroom. *J. Exp. Educ.* 1–24. 10.1080/00220973.2021.1913979

[B29] Falco A. Girardi D. Kravina L. Trifiletti E. Bartolucci G. B. Capozza D. (2013). The mediating role of psychophysic strain in the relationship between workaholism, job performance, and sickness absence: a longitudinal study. *J. Occup. Environ. Med.* 55 1255–1261. 10.1097/JOM.0000000000000007 24202241

[B30] Faragher E. B. Cass M. Cooper C. L. (2005). The relationship between job satisfaction and health: a meta-analysis. *Occup. Environ. Med.* 62 105–112. 10.1136/oem.2002.006734 15657192 PMC1740950

[B31] Fernet C. Trépanier S.-G. Demers M. Austin S. (2017). Motivational pathways of occupational and organizational turnover intention among newly registered nurses in Canada. *Nurs. Outlook* 65 444–454. 10.1016/j.outlook.2017.05.008 28641867

[B32] Fong T. C. Ho R. T. (2015). Dimensionality of the 9-item utrecht work engagement scale revisited: a bayesian structural equation modeling approach. *J. Occup. Health*, 15:0057. 10.1539/joh.15-0057-OA 25958976

[B33] Fong T. C. Ng S. (2012). Measuring engagement at work: validation of the chinese version of the utrecht work engagement scale. *Int. J. Behav. Med.* 19 391–397. 10.1007/s12529-011-9173-6 21681564 PMC3422451

[B34] Fouquereau E. Rioux L. (2002). L laboration de l’l chelle de satisfaction de vie professionnelle (I SVP) en langue fran aise une d6marche exploratoire. *Can. J. Behav. Sci.* 34 210–215.

[B35] Gignac G. E. (2016). The higher-order model imposes a proportionality constraint: that is why the bifactor model tends to fit better. *Intelligence* 55 57–68. 10.1016/j.intell.2016.01.006

[B36] Gillet N. Caesens G. Morin A. J. S. Stinglhamber F. (2019). Complementary variable- and person-centred approaches to the dimensionality of work engagement: a longitudinal investigation. *Eur. J. Work Organ. Psychol.* 28 239–258. 10.1080/1359432X.2019.1575364

[B37] Gillet N. Fouquereau E. Huyghebaert T. Colombat P. (2015). The effects of job demands and organizational resources through psychological need satisfaction and thwarting. *Span. J. Psychol.* 18:E28. 10.1017/sjp.2015.30 25991079

[B38] Gillet N. Morin A. J. S. Jeoffrion C. Fouquereau E. (2020). A person-centered perspective on the combined effects of global and specific levels of job engagement. *Group Organ. Manag.* 45 556–594.

[B39] Gillet N. Morin A. J. S. Sandrin E. Houle S. A. (2018). Investigating the combined effects of workaholism and work engagement: a substantive-methodological synergy of variable-centered and person-centered methodologies. *J. Vocat. Behav.* 109 54–77. 10.1016/j.jvb.2018.09.006

[B40] Goodboy A. K. Martin M. M. Bolkan S. (2017). Workplace bullying and work engagement: a self-determination model. *J. Interpers. Violence* 35 4686–4708. 10.1177/0886260517717492 29294812

[B41] Gorgievski M. J. Bakker A. B. Schaufeli W. B. (2010). Work engagement and workaholism: comparing the self-employed and salaried employees. *J. Posit. Psychol.* 5 83–96. 10.1080/17439760903509606

[B42] Griffiths M. D. (2005). Workaholism is still a useful construct. *Addict. Res. Theory* 13:100.

[B43] Hallberg U. E. Schaufeli W. B. (2006). “Same Same” but Different?: can work engagement be discriminated from job involvement and organizational commitment? *Eur. Psychol.* 11 119–127. 10.1027/1016-9040.11.2.119

[B44] Hardre P. L. Reeve J. (2003). A motivational model of rural students’ intentions to persist in, versus drop out of, high school. *J. Educ. Psychol.* 95 347–356. 10.1037/0022-0663.95.2.347

[B45] Ho Kim W. Park J. G. Kwon B. (2017). Work engagement in South Korea: validation of the Korean version 9-item Utrecht work engagement scale. *Psychol. Rep.* 120 561–578. 10.1177/0033294117697085 28558613

[B46] Hu L. Bentler P. M. (1999). Cutoff criteria for fit indexes in covariance structure analysis: conventional criteria versus new alternatives. *Struct. Equ. Modeling* 6 1–55. 10.1080/10705519909540118

[B47] Kenny D. A. Kaniskan B. McCoach D. B. (2015). The performance of RMSEA in models with small degrees of freedom. *Sociol. Methods Res.* 44 486–507. 10.1177/0049124114543236

[B48] Kim J. LaRose R. Peng W. (2009). Loneliness as the cause and the effect of problematic internet use: the relationship between internet use and psychological well-being. *CyberPsychol. Behav.* 12 451–455. 10.1089/cpb.2008.0327 19514821

[B49] Klassen R. M. Aldhafri S. Mansfield C. F. Purwanto E. Siu A. F. Y. Wong M. W. (2012). Teachers’ engagement at work: an international validation study. *J. Exp. Educ.* 80 317–337. 10.1080/00220973.2012.678409

[B50] Kulikowski K. (2019). One, two or three dimensions of work engagement? Testing the factorial validity of the Utrecht work engagement scale on a sample of polish employees. *Int. J. Occup. Saf. Ergon.* 25 241–249. 10.1080/10803548.2017.1371958 28849984

[B51] Kun B. Urbán R. Bõthe B. Griffiths M. D. Demetrovics Z. Kökönyei G. (2020). Maladaptive rumination mediates the relationship between self-esteem, perfectionism, and work addiction: a largescale survey study. *Int. J. Environ. Res. Public Health* 17:7332. 10.3390/ijerph17197332 33049921 PMC7579015

[B52] Lathabhavan R. Balasubramanian S. A. Natarajan T. (2017). A psychometric analysis of the Utrecht work engagement scale in Indian banking sector. *Ind. Commer. Train.* 49 296–302. 10.1108/ICT-04-2017-0031

[B53] Líbano M. D. Llorens S. Salanova M. Schaufeli W. B. (2012). About the dark and bright sides of self-efficacy: workaholism and work engagement. *Span. J. Psychol.* 15 688–701. 10.5209/rev_SJOP.2012.v15.n2.38883 22774443

[B54] Littman-Ovadia H. Balducci C. (2013). Psychometric properties of the Hebrew version of the Utrecht Work Engagement Scale (UWES-9). *Eur. J. Psychol. Assess.* 29 58–63. 10.1027/1015-5759/a000121

[B55] Littman-Ovadia H. Balducci C. Ben-Moshe T. (2014). Psychometric properties of the Hebrew Version of the Dutch Work Addiction Scale (DUWAS-10). *J. Psychol.* 148 327–346. 10.1080/00223980.2013.801334 24839730

[B56] Lovakov A. V. Agadullina E. R. Schaufeli W. B. (2017). Psychometric properties of the Russian Version of the utrecht Work engagement scale (UWES-9). *Psychol. Russ.* 10 145–162. 10.11621/pir.2017.0111

[B57] Marsh H. W. (2007). “Application of confirmatory factor analysis and structural equation modeling in sport/exercise psychology,” in *Handbook of Sport Psychology*, 3rd Edn, eds Tenenbaum G. Eklund R. C. (Hoboken, NJ: Wiley), 774–798.

[B58] Marsh H. W. Hau K.-T. Grayson D. (2005). “Goodness of fit in structural equation models,” in *Multivariate Applications Book Series. Contemporary Psychometrics: A Festschrift for Roderick P. McDonald*, eds McArdle J. J. Maydeu-Olivares A. (Mahwah, NJ: Lawrence Erlbaum Associates Publishers), 275–340.

[B59] Marsh H. W. Lüdtke O. Muthén B. Asparouhov T. Morin A. J. S. Trautwein U. (2010). A new look at the big five factor structure through exploratory structural equation modeling. *Psychol. Assess.* 22 471–491. 10.1037/a0019227 20822261

[B60] Martos T. Sallay V. Désfalvi J. Szabó T. Ittzés A. (2014). Psychometric characteristics of the Hungarian version of the Satisfaction with Life Scale (SWLS-H). *Mentálhigiéné és Pszichoszomatika* 15 289–303. 10.1556/Mental.15.2014.3.9

[B61] McDonald R. P. (1970). The theoretical foundations of principal factor analysis, canonical factor analysis, and alpha factor analysis. *Br. J. Math. Stat. Psychol.* 23 1–21. 10.1111/j.2044-8317.1970.tb00432.x

[B62] Mellor D. Stokes M. Firth L. Hayashi Y. Cummins R. (2008). Need for belonging, relationship satisfaction, loneliness, and life satisfaction. *Pers. Individ. Differ.* 45 213–218. 10.1016/j.paid.2008.03.020

[B63] Meredith W. (1993). Measurement invariance, factor analysis and factorial invariance. *Psychometrika* 58 525–543. 10.1007/BF02294825

[B64] Mills M. J. Culbertson S. S. Fullagar C. J. (2012). Conceptualizing and measuring engagement: an analysis of the Utrecht work engagement scale. *J. Happiness Stud.* 13 519–545. 10.1007/s10902-011-9277-3

[B65] Millsap R. E. (2011). *Statistical Approaches to Measurement Invariance.* Abingdon: Routledge.

[B66] Moreira-Fontán E. García-Señorán M. Conde-Rodríguez Á González A. (2019). Teachers’ ICT-related self-efficacy, job resources, and positive emotions: their structural relations with autonomous motivation and work engagement. *Comput. Educ.* 134 63–77. 10.1016/j.compedu.2019.02.007

[B67] Morin A. J. S. Arens A. K. Marsh H. W. (2016a). A bifactor exploratory structural equation modeling framework for the identification of distinct sources of construct-relevant psychometric multidimensionality. *Struct. Equ. Modeling* 23 116–139. 10.1080/10705511.2014.961800

[B68] Morin A. J. S. Boudrias J.-S. Marsh H. W. Madore I. Desrumaux P. (2016b). Further reflections on disentangling shape and level effects in person-centered analyses: an illustration exploring the dimensionality of psychological health. *Struct. Equ. Modeling* 23 438–454. 10.1080/10705511.2015.1116077

[B69] Morin A. J. S. Myers N. D. Lee S. (2020). “Modern factor analytic techniques: bifactor models, exploratory structural equation modeling (ESEM) and bifactor-ESEM,” in *Handbook of Sport Psychology*, 4th Edn, eds Tenenbaum G. Eklund R. C. (Hoboken, NJ: Wiley).

[B70] Muthén L. K. Muthén B. O. (1998). *Mplus User’s Guide*, Eighth Edn. Los Angeles, CA: Muthén & Muthén.

[B71] Nerstad C. G. L. Richardsen A. M. Martinussen M. (2009). Factorial validity of the Utrecht Work Engagement Scale (UWES) across occupational groups in Norway: factorial validity of the UWES. *Scand. J. Psychol.* 51 326–333. 10.1111/j.1467-9450.2009.00770.x 20015117

[B72] Orosz G. Dombi E. Andreassen C. S. Griffiths M. D. Demetrovics Z. (2016). Analyzing models of work addiction: single factor and bi-factor models of the bergen work addiction scale. *Int. J. Ment. Health Addict.* 14 662–671. 10.1007/s11469-015-9613-7

[B73] Panthee B. Shimazu A. Kawakami N. (2014). Validation of Nepalese version of Utrecht work engagement scale. *J. Occup. Health* 56 421–429. 10.1539/joh.14-0041-OA 25214188

[B74] Pekrun R. Murayama K. Marsh H. W. Goetz T. Frenzel A. C. (2019). Happy fish in little ponds: testing a reference group model of achievement and emotion. *J. Pers. Soc. Psychol.* 117:166. 10.1037/pspp0000230 30667258

[B75] Perreira T. A. Morin A. J. S. Hebert M. Gillet N. Houle S. A. Berta W. (2018). The short form of the Workplace Affective Commitment Multidimensional Questionnaire (WACMQ-S): a bifactor-ESEM approach among healthcare professionals. *J. Vocat. Behav.* 106 62–83. 10.1016/j.jvb.2017.12.004

[B76] Petroviæ I. B. Vukeliæ M. Èizmiæ S. (2017). Work Engagement in Serbia: psychometric properties of the Serbian Version of the Utrecht Work Engagement Scale (UWES). *Front. Psychol.* 8:1799. 10.3389/fpsyg.2017.01799 29085319 PMC5650702

[B77] Porter G. (1996). Organizational impact of workaholism: suggestions for researching the negative outcomes of excessive work. *J. Occup. Health Psychol.* 1 70–84. 10.1037/1076-8998.1.1.70 9547036

[B78] Raykov T. Penev S. (1998). Nested structural equation models: noncentrality and power of restriction test. *Struct. Equ. Modeling* 5 229–246. 10.1080/10705519809540103

[B79] Reise S. P. (2012). The rediscovery of bifactor measurement models. *Multivar. Behav. Res.* 47 667–696. 10.1080/00273171.2012.715555 24049214 PMC3773879

[B80] Ryan R. M. Deci E. L. (2001). On happiness and human potentials: a review of research on hedonic and eudaimonic well-being. *Annu. Rev. Psychol.* 52 141–166. 10.1146/annurev.psych.52.1.141 11148302

[B81] Ryan R. M. Deci E. L. (2017). *Self-Determination Theory: Basic Psychological Needs in Motivation, Development, and Wellness.* New York, NY: Guilford Press.

[B82] Salanova M. Del Líbano M. Llorens S. Schaufeli W. B. (2014). Engaged, workaholic, burned-out or just 9-to-5? toward a typology of employee well-being: employee well-being and work investment. *Stress Health* 30 71–81. 10.1002/smi.2499 23723156

[B83] Schaufeli W. B. (2018). Work engagement in Europe: relations with national economy, governance, and culture. *Organ. Dyn.* 47 99–106. 10.1016/j.orgdyn.2018.01.003

[B84] Schaufeli W. B. Bakker A. B. Salanova M. (2006). The measurement of work engagement with a short questionnaire: a cross-national study. *Educ. Psychol. Meas.* 66 701–716. 10.1177/0013164405282471

[B85] Schaufeli W. B. Bakker A. B. van der Heijden F. M. M. A. Prins J. T. (2009). Workaholism, burnout and well-being among junior doctors: the mediating role of role conflict. *Work Stress* 23 155–172. 10.1080/02678370902834021

[B86] Schaufeli W. B. Salanova M. González-Romá V. Bakker A. B. (2002). The measurement of engagement and burnout: a two sample confirmatory factor analytic approach. *Journal Happiness Stud.* 3 71–92.

[B87] Schaufeli W. B. Shimazu A. Hakanen J. Salanova M. De Witte H. (2019). An ultra-short measure for work engagement: the UWES-3 validation across five countries. *Eur. J. Psychol. Assess.* 35 577–591. 10.1027/1015-5759/a000430

[B88] Schaufeli W. B. Taris T. W. van Rhenen W. (2008). Workaholism, burnout, and work engagement: three of a kind or three different kinds of employee well-being? *Appl. Psychol.* 57 173–203. 10.1111/j.1464-0597.2007.00285.x

[B89] Seppälä P. Mauno S. Feldt T. Hakanen J. Kinnunen U. Tolvanen A. (2009). The construct validity of the Utrecht work engagement scale: multisample and longitudinal evidence. *J. Happiness Stud.* 10 459–481. 10.1007/s10902-008-9100-y

[B90] Shimazu A. Schaufeli W. B. Kamiyama K. Kawakami N. (2015). Workaholism vs. Work Engagement: the two different predictors of future well-being and performance. *Int. J. Behav. Med.* 22 18–23. 10.1007/s12529-014-9410-x 24696043

[B91] Shuck B. Zigarmi D. Owen J. (2015). Psychological needs, engagement, and work intentions: a Bayesian multi-measurement mediation approach and implications for HRD. *Eur. J. Train. Dev.* 39 2–21. 10.1108/EJTD-08-2014-0061

[B92] Simbula S. Guglielmi D. Schaufeli W. B. Depolo M. (2013). An Italian validation of the Utrecht work engagement scale: characterization of engaged groups in a sample of schoolteachers. *Appl. Psychol. Bull.* 61 43–54.

[B93] Sinval J. Pasian S. Queirós C. Marôco J. (2018). Brazil-Portugal transcultural adaptation of the UWES-9: internal consistency, dimensionality, and measurement invariance. *Front. Psychol.* 9:353. 10.3389/fpsyg.2018.00353 29618995 PMC5872586

[B94] Tóth-Király I. Bõthe B. Márki A. N. Rigó A. Orosz G. (2019a). Two sides of the same coin: the differentiating role of need satisfaction and frustration in passion for screen-based activities. *Eur. J. Soc. Psychol.* 49 1190–1205. 10.1002/ejsp.2588

[B95] Tóth-Király I. Bõthe B. Orosz G. Rigó A. (2019b). A new look on the representation and criterion validity of need fulfillment: application of the bifactor exploratory structural equation modeling framework. *J. Happiness Stud.* 20 1609–1626. 10.1007/s10902-018-0015-y

[B96] Tóth-Király I. Gajdos P. Román N. Vass N. Rigó A. (2019c). The associations between orthorexia nervosa and the sociocultural attitudes: the mediating role of basic psychological needs and health anxiety. *Eat. Weight Disord.* 26 125–134. 10.1007/s40519-019-00826-1 31811515

[B97] Tóth-Király I. Morin A. J. Salmela-Aro K. (2021). A longitudinal perspective on the associations between work engagement and workaholism. *Work Stress* 35 27–56. 10.1080/02678373.2020.1801888

[B98] Tóth-Király I. Morin A. J. S. Bõthe B. Orosz G. Rigó A. (2018). Investigating the multidimensionality of need fulfillment: a bifactor exploratory structural equation modeling representation. *Struct. Equ. Modeling* 25 267–286. 10.1080/10705511.2017.1374867

[B99] Tóth-Király I. Morin A. J. Bõthe B. Rigó A. Orosz G. (2020). Toward an improved understanding of work motivation profiles. *Appl. Psychol.* 70 986–1017. 10.1111/apps.12256

[B100] Trépanier S.-G. Fernet C. Austin S. (2013). Workplace bullying and psychological health at work: the mediating role of satisfaction of needs for autonomy, competence and relatedness. *Work Stress* 27 123–140. 10.1080/02678373.2013.782158

[B101] Trépanier S.-G. Fernet C. Austin S. (2015). A longitudinal investigation of workplace bullying, basic need satisfaction, and employee functioning. *J. Occup. Health Psychol.* 20 105–116. 10.1037/a0037726 25151460

[B102] Urbán R. Kun B. Mózes T. Soltész P. Paksi B. Farkas J. (2019). A four-factor model of work addiction: the development of the work addiction risk test revised. *Eur. Addict. Res.* 25 145–160. 10.1159/000499672 30982051

[B103] Vallerand R. J. Fortier M. S. Guay F. (1997). Self-determination and persistence in a real-life setting: toward a motivational model of high school dropout. *J. Pers. Soc. Psychol.* 72 1161–1176. 10.1037//0022-3514.72.5.1161 9150590

[B104] Vallières F. McAuliffe E. Hyland P. Galligan M. Ghee A. (2017). Measuring work engagement among community health workers in Sierra Leone: validating the Utrecht Work Engagement Scale. *Rev. Psicol. Trab. y de Las Organ.* 33 41–46. 10.1016/j.rpto.2016.12.001

[B105] van Beek I. Hu Q. Schaufeli W. B. Taris T. W. Schreurs B. H. J. (2012). For fun, love, or money: what drives workaholic, engaged, and burned-out employees at work? *Appl. Psychol.* 61 30–55. 10.1111/j.1464-0597.2011.00454.x

[B106] Van den Berghe L. Cardon G. Tallir I. Kirk D. Haerens L. (2016). Dynamics of need-supportive and need-thwarting teaching behavior: the bidirectional relationship with student engagement and disengagement in the beginning of a lesson. *Phys. Educ. Sport Pedagogy* 21 653–670. 10.1080/17408989.2015.1115008

[B107] Van den Broeck A. Ferris D. L. Chang C.-H. Rosen C. C. (2016). A review of self-determination theory’s basic psychological needs at work. *J. Manag.* 42 1195–1229. 10.1177/0149206316632058

[B108] Vansteenkiste M. Ryan R. M. (2013). On psychological growth and vulnerability: basic psychological need satisfaction and need frustration as a unifying principle. *J. Psychother. Integr.* 23 263–280. 10.1037/a0032359

[B109] Vazquez A. C. S. Magnan E. dos S. Pacico J. C. Hutz C. S. Schaufeli W. B. (2015). Adaptation and validation of the Brazilian version of the Utrecht work engagement scale. *Psico-USF* 20 207–217. 10.1590/1413-82712015200202

[B110] Villotti P. Balducci C. Zaniboni S. Corbière M. Fraccaroli F. (2014). An analysis of work engagement among workers with mental disorders recently integrated to work. *J. Career Assess.* 22 18–27. 10.1177/1069072713487500

[B111] Wang Z. Chen L. Duan Y. Du J. (2018). Supervisory mentoring and newcomers’ work engagement: the mediating role of basic psychological need satisfaction. *Soc. Behav. Pers.* 46 1745–1760. 10.2224/sbp.7609

[B112] Wefald A. J. Mills M. J. Smith M. R. Downey R. G. (2012). A comparison of three job engagement measures: examining their factorial and criterion-related validity: comparison of engagement measures. *Appl. Psychol.* 4 67–90. 10.1111/j.1758-0854.2011.01059.x 26286971

[B113] Yusoff R. B. Ali A. Khan A. Bakar S. A. (2013). Psychometric evaluation of Utrecht work engagement scale among academic staff in universities of Pakistan. *World Appl. Sci. J.* 28 1555–1560. 10.5829/idosi.wasj.2013.28.11.2014

[B114] Zecca G. Györkös C. Becker J. Massoudi K. de Bruin G. P. Rossier J. (2015). Validation of the French Utrecht work engagement scale and its relationship with personality traits and impulsivity. *Eur. Rev. Appl. Psychol.* 65 19–28. 10.1016/j.erap.2014.10.003

[B115] Zeijen M. E. L. Peeters M. C. W. Hakanen J. J. (2018). Workaholism versus work engagement and job crafting: what is the role of self-management strategies? *Hum. Resour. Manag. J.* 28 357–373. 10.1111/1748-8583.12187

