# Supplementary material for: Having the Cake and Eating It Too: First-Order, Second-Order and Bifactor Representations of Work Engagement
Source: Front Psychol. 2021 Jul 22;12:615581. doi: 10.3389/fpsyg.2021.615581 (PMC8339798; doi:10.3389/fpsyg.2021.615581)
Supplement: Supplementary file 1 [file Table_1.DOCX]

***Online supplementary materials for:***

**Having the Cake and Eating It Too: Investigating the Global and Specific Nature of Work Engagement**

**Table of Contents:**

Appendix 1: Hungarian and original English version of the short version of Utrecht Work Engagement Scale (UWES-9)

Appendix 2: Preliminary Measurement Models

Table S1: Prior validity and reliability characteristics of the 9 item Utrecht Work Engagement Scale

Table S2: Parameter Estimates from the Correlates Measurement Model Estimated in Sample 1

Table S3: Parameter Estimates from the Correlates Measurement Model Estimated in Sample 2

**Appendix 1**

**Hungarian and original English version of the short version of Utrecht Work Engagement Scale (UWES-9)**

|  | **Hungarian Version** | **English Version (Schaufeli et al., 2006)** |
| --- | --- | --- |
| Instructions | Az alábbiakban 9 állítást fogsz olvasni a munkával kapcsolatos érzéseidről. Olvass el minden állítást figyelmesen és döntsd el, hogy tapasztaltad-e már az adott érzelmet a munkád során és ha igen, akkor milyen gyakran! | The following 9 statements are about how you feel at work. Please read each statement carefully and decide if you ever feel this way about your job. Indicate how often you felt it by crossing the number that best describes how frequently you feel that way. |
| Rating Scale | 1 – Soha  2 – Szinte soha  3 – Ritkán  4 – Néha  5 – Gyakran  6 – Nagyon gyakran  7 – Mindig | 1 – Never  2 – Almost never  3 – Rarely  4 – Sometimes  5 – Often  6 – Very Often  7 – Always |
| Item 1 (Vigor) | A munkahelyemen tele vagyok energiával. | At my work, I feel bursting with energy. |
| Item 2 (Vigor) | A munkámban erőteljesnek és élénknek érzem magam. | At my job, I feel strong and vigorous. |
| Item 3 (Dedication) | Lelkesedem a munkámért. | I am enthusiastic about my job. |
| Item 4 (Dedication) | A munkám inspirál. | My job inspires me. |
| Item 5 (Vigor) | Reggelente van kedvem dolgozni menni. | When I get up in the morning, I feel like going to work. |
| Item 6 (Absorption) | Boldog vagyok, amikor elmélyülten dolgozom. | I feel happy when I am working intensely. |
| Item 7 (Dedication) | Büszke vagyok a munkára, amit végzek. | I am proud of the work that I do. |
| Item 8 (Absorption) | Belemerülök a munkámba. | I am immersed in my work. |
| Item 9 (Absorption) | A munkám teljesen magával ragad. | I get carried away when I am working. |

**Appendix 2**

**Preliminary Measurement Models**

Preliminary analyses were carried out to examine the psychometric properties of the measures assessing the correlates, and to derive factor scores from these measurement models to examine the criterion validity of the most optimal representation of work engagement.

**Model Estimation**

All analyses were conducted with Mplus 8 (Muthén & Muthén, 2017), and, similar to the main study, models were estimated with the robust maximum likelihood (MLR) estimator. The adequacy of the models was evaluated based on commonly-reported sample size-independent goodness-of-fit indices (Hu & Bentler, 1999; Marsh et al., 2005): the comparative fit index (CFI), the Tucker-Lewis Index (TLI), and the root mean square error of approximation (RMSEA). CFI and TLI were considered adequate or excellent when their values were higher than .90 and .95, respectively. Conversely RMSEA was considered acceptable and excellent when it had a value smaller than .08 and .06, respectively. McDonald’s (1970) model-based composite reliability coefficient (ω) was also calculated as an indicator of reliability.

**Sample 1.** With respect to basic psychological need fulfillment, our decision to rely on the bifactor exploratory structural equation modeling (bifactor-ESEM; Morin, Arens, & Marsh, 2016; Morin, Arens, Tran, et al., 2016) framework is based on recent evidence showing that need fulfillment was best represented using this analytical framework, and that there is an added value of relying on ESEM (Myers et al., 2014; Tóth-Király, Bőthe, et al., 2018), bifactor (Brunet et al., 2016; Gillet et al., 2019), or bifactor-ESEM (Sánchez-Oliva et al., 2017; Tóth-Király et al., 2019; Tóth-Király, Morin, et al., 2018) components. In bifactor-ESEM, the ESEM component entails the free estimation of all cross-loadings between items and all factors (instead of forcing them to be zero). Recent statistical research has already shown that the free estimation of cross-loadings results in a more accurate depiction of the latent constructs even when very small (i.e., .100) cross-loadings are present in the population model, but, at the same time, the measurement model remains unbiased when no cross-loadings are present in the population model (for a review, see Asparouhov, Muthén, & Morin, 2015). The bifactor component allows the estimation of a global (G-) factor reflecting global levels of need fulfillment at work, while also taking into account the unique qualities associated with each of the specific psychological needs that is not explained by the G-factor in the form of uncorrelated specific (S-) factors. As for turnover intention, it was operationalized as a unidimensional construct, thus modelled following the standard CFA specification where all items were specified to load on a single latent factor.

**Sample 2.** Work addiction and work satisfaction are thought to be relatively distinct construct and were thus measured with two separate scales. For this reason, they were estimated as a two-factor CFA model representing work addiction and work satisfaction with one a priori correlate uniqueness between the first two work addiction items.

**Results**

**Sample 1.** The measurement model in Sample 1 demonstrated good fit (χ^2^ = 279.155, df = 194; CFI = .966, TLI = .938, RMSEA = .043 [90% CI .031, .053]). Parameter estimates associated with this model are reported in Table S2. Examination of these estimates revealed a well-defined and reliable global need fulfillment factor (|λ| = .041 to .652, *M* = .513, ω = .933). As for the specific need fulfillment factors, relatedness satisfaction (|λ| = .319 to .704, *M* = .529, ω = .727) and autonomy frustration (|λ| = .229 to .673, *M* = .420, ω = .665) appeared to have retained a higher amount of specificity over and above the global factor. By contrast, autonomy satisfaction (|λ| = .383 to .545, *M* = .438, ω = .584), competence satisfaction (|λ| = .039 to .547, *M* = .392, ω = .570), and relatedness frustration (|λ| = .191 to .813, *M* = .378, ω = .561) retained a moderate amount of specificity, whereas competence frustration retained a lower amount of specificity (|λ| = .067 to .417, *M* = .183, ω = .225). Finally, turnover intention was also well-defined and highly reliable (|λ| = .820 to .956, *M* = .906, ω = .933).

**Sample 2.** Results pertaining to the preliminary measurement model were similar to Sample 1 in that the fit of the measurement model was adequate (χ^2^ = 164.904, df = 42; CFI = .926, TLI = .904, RMSEA = .076 [90% CI .064, .089]). Both the work addiction (|λ| = .466 to .676, *M* = .571, ω = .745) and work satisfaction (|λ| = .608 to .903, *M* = .763, ω = .879) factors were well-defined and highly reliable. Factor scores were derived from these measurement models and used in the main study.

**References**

Asparouhov, T., Muthén, B., & Morin, A.J.S. (2015). Bayesian Structural equation modeling with cross-loadings and residual covariances: Comments on Stromeyer et al. *Journal of Management*, *41*, 1561-1577

Balducci, C., Fraccaroli, F., & Schaufeli, W. B. (2010). Psychometric properties of the Italian Version of the Utrecht Work Engagement Scale (UWES-9): A cross-cultural analysis. European Journal of Psychological Assessment, 26(2), 143–149.

Breevaart, K., Bakker, A. B., Demerouti, E., & Hetland, J. (2012). The measurement of state work engagement: A multilevel factor analytic study. European Journal of Psychological Assessment, 28(4), 305–312.

Brunet, J., Gunnel, K.E., Teixeira, P., Sabiston, C.M., & Bélanger, M. (2016). Should we be looking at the forest or the trees? Overall psychological need satisfaction and individual needs as predictors of physical activity. *Journal of Sport & Exercise Psychology, 38*, 317–330

Chaudhary, R., Rangnekar, S., & Barua, M. K. (2012). Psychometric evaluation of Utrecht Work Engagement Scale in an Indian sample. Asia-Pacific Journal of Management Research and Innovation, 8(3), 343–350.

de Bruin, G. P., & Henn, C. M. (2013). Dimensionality of the 9-Item Utrecht Work Engagement Scale (UWES-9). Psychological Reports, 112(3), 788–799.

Fong, T. C., & Ho, R. T. H. (2015). Dimensionality of the 9‐item Utrecht Work Engagement Scale revisited: A Bayesian structural equation modeling approach. Journal of Occupational Health, 57(4), 353–358.

Fong, T. C., & Ng, S. (2012). Measuring Engagement at Work: Validation of the Chinese Version of the Utrecht Work Engagement Scale. International Journal of Behavioral Medicine, 19(3), 391–397.

Gillet, N., Morin, A.J.S., Huyghebaert-Zouaghi, T., Alibran, E., Barrault, S., & Vanhove‐Meriaux, C. (2019). Students’ Need Satisfaction Profiles: Similarity and Change over the Course of a University Semester. *Applied Psychology*.

Hallberg, U. E., & Schaufeli, W. B. (2006). “Same Same” but Different?: Can Work Engagement Be Discriminated from Job Involvement and Organizational Commitment? European Psychologist, 11(2), 119–127.

Ho Kim, W., Park, J. G., & Kwon, B. (2017). Work Engagement in South Korea: Validation of the Korean Version 9-Item Utrecht Work Engagement Scale. Psychological Reports, 120(3), 561–578.

Hu, L., & Bentler, P.M. (1999). Cutoff criteria for fit indexes in covariance structure analysis: Conventional criteria versus new alternatives. *Structural Equation Modeling, 6*, 1-55.

Klassen, R. M., Aldhafri, S., Mansfield, C. F., Purwanto, E., Siu, A. F. Y., Wong, M. W., & Woods-McConney, A. (2012). Teachers’ engagement at work: An international validation study. The Journal of Experimental Education, 80(4), 317–337.

Kulikowski, K. (2019). One, two or three dimensions of work engagement? Testing the factorial validity of the Utrecht Work Engagement Scale on a sample of Polish employees. International Journal of Occupational Safety and Ergonomics, 25(2), 241–249.

Lathabhavan, R., Balasubramanian, S. A., & Natarajan, T. (2017). A psychometric analysis of the Utrecht Work Engagement Scale in Indian banking sector. Industrial and Commercial Training, 49(6), 296–302.

Littman-Ovadia, H., & Balducci, C. (2013). Psychometric properties of the Hebrew Version of the Utrecht Work Engagement Scale (UWES-9). European Journal of Psychological Assessment, 29(1), 58–63.

Lovakov, A. V., Agadullina, E. R., & Schaufeli, W. B. (2017). Psychometric properties of the Russian Version of the utrecht Work engagement scale (UWES-9). Psychology in Russia: State of the Art, 10(1), 145–162.

Marsh, H.W., Hau, K., & Grayson, D. (2005). Goodness of fit in structural equation models. In A. Maydeu-Olivares & J. McArdle (Eds.), *Contemporary psychometrics* (pp. 275-340). Mahwah, NJ: Erlbaum.

McDonald, R.P. (1970). Theoretical foundations of principal factor analysis, canonical factor analysis, and alpha factor analysis. *British Journal of Mathematical & Statistical Psychology*, *23*, 1-21.

Mills, M. J., Culbertson, S. S., & Fullagar, C. J. (2012). Conceptualizing and measuring Engagement: An analysis of the Utrecht Work Engagement Scale. Journal of Happiness Studies, 13(3), 519–545.

Moreira-Fontán, E., García-Señorán, M., Conde-Rodríguez, Á., & González, A. (2019). Teachers’ ICT-related self-efficacy, job resources, and positive emotions: Their structural relations with autonomous motivation and work engagement. Computers & Education, 134, 63–77.

Morin, A.J.S., Arens, A., & Marsh, H. (2016a). A bifactor exploratory structural equation modeling framework for the identification of distinct sources of construct-relevant psychometric multidimensionality. *Structural Equation Modeling*, *23*, 116-139.

Morin, A.J.S., Arens, K., Tran, A., & Caci, H. (2016b). Exploring sources of construct-relevant multidimensionality in psychiatric measurement: A tutorial and illustration using the Composite Scale of Morningness. *International Journal of Methods in Psychiatric Research*, *25*, 277-288.

Muthén, L.K., & Muthén, B.O. (2017). *Mplus user guide.* Los Angeles, CA: Muthén & Muthén.

Myers, N.D., Martin, J.J., Ntoumanis, N., Celimli, S., & Bartholomew, K.J. (2014). Exploratory bifactor analysis in sport, exercise, and performance psychology: A substantive-methodological synergy. *Sport, Exercice, & Performance Psychology, 3*, 258–272.

Nerstad, C. G. L., Richardsen, A. M., & Martinussen, M. (2009). Factorial validity of the Utrecht Work Engagement Scale (UWES) across occupational groups in Norway: Factorial validity of the UWES. Scandinavian Journal of Psychology.

Panthee, B., Shimazu, A., & Kawakami, N. (2014). Validation of Nepalese Version of Utrecht Work Engagement Scale. Journal of Occupational Health, 56(6), 421–429.

Petrović, I. B., Vukelić, M., & Čizmić, S. (2017). Work Engagement in Serbia: Psychometric properties of the Serbian Version of the Utrecht Work Engagement Scale (UWES). Frontiers in Psychology, 8, 1799.

Sánchez-Oliva, D., Morin, A.J.S., Teixeira, P.J., Carraça, E.V., Palmeira, A.L., & Silva, M.N. (2017). A bifactor-exploratory structural equation modeling representation of the structure of basic psychological needs at work scale. *Journal of Vocational Behavior*, *98*, 173-187.

Schaufeli, W. B., Bakker, A. B., & Salanova, M. (2006). The measurement of work engagement with a short questionnaire: A cross-national study. Educational and Psychological Measurement, 66(4), 701–716.

Seppälä, P., Mauno, S., Feldt, T., Hakanen, J., Kinnunen, U., Tolvanen, A., & Schaufeli, W. (2009). The construct validity of the Utrecht Work Engagement Scale: Multisample and longitudinal evidence. Journal of Happiness Studies, 10(4), 459–481.

Simbula, S., Guglielmi, D., Schaufeli, W. B., & Depolo, M. (2013). An Italian validation of the Utrecht Work Engagement Scale: Characterization of engaged groups in a sample of schoolteachers. Applied Psychology Bulletin, 61(268), 43–54.

Sinval, J., Pasian, S., Queirós, C., & Marôco, J. (2018). Brazil-Portugal transcultural adaptation of the UWES-9: Internal consistency, dimensionality, and measurement invariance. Frontiers in Psychology, 9, 353.

Tóth-Király, I., Bőthe, B., Orosz, G., & Rigó, A. (2018). On the importance of balanced need fulfillment: A person-centered perspective. *Journal of Happiness Studies*, 1-22.

Tóth-Király, I., Bőthe, B., Orosz, G., & Rigó, A. (2019). A new Look on the representation and criterion validity of need fulfillment: Application of the bifactor exploratory structural equation modeling framework. *Journal of Happiness Studies, 20*, 1609-1626.

Tóth-Király, I., Morin, A.J.S., Bőthe, B., Orosz, G., & Rigó, A. (2018). Investigating the multidimensionality of need fulfillment: A bifactor exploratory structural equation modeling representation. *Structural Equation Modeling, 25*, 267-286.

Vallières, F., McAuliffe, E., Hyland, P., Galligan, M., & Ghee, A. (2017). Measuring work engagement among community health workers in Sierra Leone: Validating the Utrecht Work Engagement Scale. Revista de Psicología Del Trabajo y de Las Organizaciones, 33(1), 41–46.

Vazquez, A. C. S., Magnan, E. dos S., Pacico, J. C., Hutz, C. S., & Schaufeli, W. B. (2015). Adaptation and Validation of the Brazilian Version of the Utrecht Work Engagement Scale. Psico-USF, 20(2), 207–217.

Villotti, P., Balducci, C., Zaniboni, S., Corbière, M., & Fraccaroli, F. (2014). An analysis of Work Engagement among workers with mental disorders recently integrated to work. Journal of Career Assessment, 22(1), 18–27.

Wefald, A. J., Mills, M. J., Smith, M. R., & Downey, R. G. (2012). A comparison of three job engagement measures: Examining their factorial and criterion-related validity: Comparison of engagement measures. Applied Psychology: Health and Well-Being, 4(1), 67–90.

Yusoff, R. B., Ali, A., Khan, A., & Bakar, S. A. (2013). Psychometric evaluation of Utrecht Work Engagement Scale among academic staff in universities of Pakistan. World Applied Sciences Journal, 28(11), 1555–1560.

Zecca, G., Györkös, C., Becker, J., Massoudi, K., de Bruin, G. P., & Rossier, J. (2015). Validation of the French Utrecht Work Engagement Scale and its relationship with personality traits and impulsivity. European Review of Applied Psychology, 65(1), 19–28.

Zeijen, M. E. L., Peeters, M. C. W., & Hakanen, J. J. (2018). Workaholism versus work engagement and job crafting: What is the role of self-management strategies? Human Resource Management Journal, 28(2), 357–373.

**Table S1**

*Prior validity and reliability characteristics of the 9 item Utrecht Work Engagement Scale†*

| Authors | Nation | Sample | Analysis | Vigor (Cronbach’s α) | Dedication (Cronbach’s α) | Absorption (Cronbach’s α) | χ^2^ | df | CFI | TLI | RMSEA | Corr |
| --- | --- | --- | --- | --- | --- | --- | --- | --- | --- | --- | --- | --- |
| Balducci et al. (2010) - study 1 | Italy | N = 668  M_age_ = NA | 3-factor CFA^b^ | .86 | .89 | .76 | 58.600 | 20 | .982 | — | .077 | .70 |
| Balducci et al. (2010) - study 2 | The Netherlands | N = 2,213 M_age_ = 34.9 | 3-factor CFA^b^ | — | — | — | 175.300 | 22 | .973 | — | .080 | — |
| Breevaart et al. (2012) | The Netherlands | N = 271  M_age_ = 36.75 | 3-factor CFA^a^ | — | — | — | 317.400 | 48 | .960 | — | .060 | .94 |
| Chaudhary et al. (2012) | India | N = 438  M_age_ = 33.24 | 3-factor CFA^b^ | .60 | .65 | .59 | 45.530 | 23 | .976 | .963 | .047 | .95 |
| de Bruin & Henn (2013) | South Africa | N = 369  M_age_ = 35.3 | partial Bifactor-CFA | — | — | — | 38.763 | 23 | .993 | .996 | .044 | — |
| Fong & Ng (2012) | Hong Kong | N = 992  M_age_ = 43.2 | 3-factor CFA^a^ | .74 | .77 | .70 | 172.270 | 24 | .930 | .900 | .080 | .78-.95 |
| Fong & Ho (2015) | Hong Kong | N = 1,112  M_age_ = NA | partial Bifactor-CFA | — | — | — | 86.400 | 33 | .945 | — | .075 | — |
| Hallberg & Schaufeli (2006)* | Sweden | N = 186  M_age_ = 41 | 3-factor CFA^c^ | .85 | .89 | .76 | 93.870 | 24 | .970 | — | .130 | .92 |
| Hallberg & Schaufeli (2006)* | Sweden | N = 186  M_age_ = 41 | 1-factor CFA | .85 | .89 | .76 | 111.140 | 27 | .970 | — | .130 |  |
| Ho Kim et al. (2017) - study 1 | South Korea | N = 307  M_age_ = 39.2 | 3-factor EFA | .92 | .90 | .91 | 34.801 | 12 | .985 | .954 | .079 | — |
| Ho Kim et al. (2017) - study 2 | South Korea | N = 342  M_age_ = 37.6 | 3-factor CFA^c^ | .91 | .89 | .90 | 92.528 | 24 | .966 | .949 | .091 | .77 |
| Klassen et al. (2012) | Combined groups | N = 856  M_age_ = NA | 1-factor CFA | — | — | — | 267.760 | 126 | .970 | — | .040 | — |
| Kulkowski (2019) | Poland | N = 1420  M_age_ = NA | 3-factor CFA^c^ | .85 | .79 | .77 | 497.200 | 24 | .940 | — | .120 | .71 |
| Lathabhavan et al. (2017) | India | N = 467  M_age_ = 38 | 3-factor CFA^a^ | .90 | .89 | .95 | 45.740 | 24 | .990 | .990 | .040 | .44 |
| Littman-Ovadia & Balducci (2013) | Israel | N = 252  M_age_ = 33.7 | 3-factor CFA^c^ | .85 | .86 | .84 | 67.471 | 24 | .988 | — | .085 | .87 |
| Lovakov et al. (2017) | Russia | N = 1,783  M_age_ = 36.36 | 3-factor CFA^b^ | .79 | .87 | .75 | 319.730 | 22 | .950 | .920 | .090 | .73 |
| Mills et al. (2012) - study 1 | USA | N = 98  M_age_ = 41.06 | 3-factor CFA^c^ | .83 | .84 | .70 | 46.320 | 24 | .950 | — | .100 | .66 |
| Mills et al. (2012) - study 2 | USA | N = 120  M_age_ = 39 | 1-factor CFA | .76 | .48 | .49 | 55.910 | 27 | .940 | — | .090 | .71 |
| Moreira-Fontán et al. (2019) | Spain | N = 350  M_age_ = 48.40 | 3-factor CFA^a^ | .83 | .85 | .83 | — | — | .982 | — | .074 | .78 |
| Nerstad et al. (2010) | Norway | N = 1,266  M_age_ = 40.8 | 3-factor CFA^a^ | .65 | .84 | .83 | 178.420 | 24 | .990 | — | .070 | .86 |
| Panthee et al. (2014) | Nepal | N = 438  M_age_ = 30.85 | 3-factor CFA^a^ | .60 | .78 | .76 | 90.110 | 24 | .950 | .930 | .070 | .57 |
| Petrovic et al. (2017) | Serbia | N = 860  M_age_ = 40 | 3-factor CFA^c^ | .85 | .87 | .62 | 116.546 | 24 | .868 | — | .067 | .70 |
| Schaufeli et al. (2006) | 10 countries | N = 14,521  M_age_ = 40.3 | 3-factor CFA^a^ | .77 | .85 | .78 | 3227.290 | 240 | .960 | — | .030 | .92 |
| Seppälä et al. (2009) | Finland | N = 9,404  M_age_ = NA | 3-factor CFA^a^ | — | — | — | 1328.650 | 120 | .980 | — | .076 | .83-.97 |
| Simbula et al. (2013) | Italy | N = 488  M_age_ = NA | 3-factor CFA^b^ | .80 | .85 | .79 | 94.910 | 22 | .970 | — | .080 | .77 |
| Sinval et al. (2018) - study 1 | Brazil and Portugal | N = 1,046  M_age_ = 35.57 | Second-order CFA | .93 | .93 | .90 | 409.919 | 25 | .998 | .997 | .121 | — |
| Sinval et al. (2018) - study 2 | Portugal | N = 3,623  M_age_ = 33.35 | Second-order CFA | .90 | .91 | .82 | 498.849 | 24 | .998 | .997 | .074 | — |
| Vallières et al. (2017) | Sierra Leone | N = 323  M_age_ = NA | 1-factor CFA | — | — | — | 49.121 | 27 | .911 | .882 | .050 | — |
| Vazquez et al. (2015) | Brazil | N = 1,167  M_age_ = 36.8 | 3-factor CFA^c^ | — | — | — | 472.790 | 66 | .980 | .980 | .120 | — |
| Villotti et al. (2014) | Italy | N = 310  M_age_ = 41.17 | 3-factor CFA^c^ | .86 | .90 | .85 | 75.710 | 24 | .986 | — | .092 | .82 |
| Wefald et al. (2012) | USA | N = 382  M_age_ = NA | 3-factor CFA^c^ | .87 | .84 | .78 | 250.150 | 24 | .910 | — | .160 | .75 |
| Yusoff et al. (2013) | Pakistan | N = 400  M_age_ = NA | 3-factor CFA^a^ | .87 | .84 | .90 | 25.300 | 9 | .990 | — | .064 | .63 |
| Zecca et al. (2014) | Switzerland | N = 661  M_age_ = 40.86 | 3-factor CFA^b^ | .81 | .90 | .82 | 92.910 | 21 | .980 | .970 | .070 | .71 |
| Zeijen et al. (2018) | The Netherlands | N = 372  M_age_ = 40.75 | 3-factor CFA^c^ | .88 | .91 | .83 | 183.265 | 23 | .940 | — | .156 | .78 |

*Note*. CFA = confirmatory factor analysis; ^a^ Good fit without manipulation; ^b^ Modified; ^c^ Unsatisfactory fit indices without manipulation; * Authors accepted both the 3-factor and the 1-factor models as final models.

**Table S2**

*Parameter Estimates from the Correlates Measurement Model Estimated in Sample 1*

|  | NF (λ) | AS (λ) | RS (λ) | CS (λ) | AF (λ) | RF (λ) | CF (λ) | TI (λ) | δ |
| --- | --- | --- | --- | --- | --- | --- | --- | --- | --- |
| Autonomy satisfaction (AS) |  |  |  |  |  |  |  |  |  |
| Item 1 | **.514**** | **.398**** | .067 | -.100 | -.079 | .088 | .026 |  | .549 |
| Item 7 | **.548**** | **.383**** | -.034 | .176* | .081 | .116 | .019 |  | .822 |
| Item 13 | **.588**** | **.545**** | .075 | .113 | -.031 | .040 | -.041 |  | .673 |
| Item 19 | **.559**** | **.425**** | .139 | -.085 | -.159* | -.061 | -.080 |  | .142 |
| Relatedness satisfaction (RS) |  |  |  |  |  |  |  |  |  |
| Item 3 | **.454**** | .070 | **.319**** | -.082 | -.059 | -.063 | .018 |  | .378 |
| Item 9 | **.397**** | .075 | **.704*** | -.014 | .069 | .026 | .161 |  | .400 |
| Item 15 | **.387**** | .079 | **.683**** | .072 | .029 | -.196** | -.030 |  | .500 |
| Item 21 | **.508**** | -.036 | **.411**** | -.258** | -.004 | -.130 | -.068 |  | .401 |
| Competence satisfaction (CS) |  |  |  |  |  |  |  |  |  |
| Item 5 | **.533**** | -.131 | -.108 | **.511**** | .032 | .123 | .180 |  | .310 |
| Item 11 | **.351**** | .286** | .003 | **.547**** | .104 | .054 | .013 |  | .534 |
| Item 17 | **.620**** | .210 | -.074 | **.039** | -.039 | .030 | -.145 |  | .482 |
| Item 23 | **.514** | -.094 | -.077 | **.470**** | .039 | .059 | -.303 |  | .526 |
| Autonomy frustration (AF) |  |  |  |  |  |  |  |  |  |
| Item 2 | **.041** | .087 | -.041 | -.121 | **.372*** | .074 | .093 |  | .335 |
| Item 8 | **-.623**** | -.082 | .066 | .131* | **.407** | .006 | -.131 |  | .318 |
| Item 14 | **-.470**** | -.005 | .057 | .061 | **.673**** | -.013 | .023 |  | .332 |
| Item 20 | **-.572**** | -.353* | -.007 | .176* | **.229** | .064 | -.091 |  | .439 |
| Relatedness frustration (RF) |  |  |  |  |  |  |  |  |  |
| Item 4 | **-.428**** | .023 | -.069 | .001 | .085 | **.813**** | -.033 |  | .541 |
| Item 10 | **-.573**** | .190 | -.168 | .160 | .050 | **.191** | -.094 |  | .356 |
| Item 16 | **-.644**** | .061 | -.082 | .179** | -.095 | **.307**** | .037 |  | .444 |
| Item 22 | **-.584**** | .006 | -.376** | .171** | -.068 | **.202*** | .070 |  | .453 |
| Competence frustration (CF) |  |  |  |  |  |  |  |  |  |
| Item 6 | **-.534**** | .254 | .290 | -.359** | -.094 | .053 | **-.162** |  | .484 |
| Item 12 | **-.652**** | .081 | .166 | -.087 | -.030 | -.050 | **.067** |  | .438 |
| Item 18 | **-.619**** | -.011 | .197 | -.207 | -.068 | -.019 | **.417** |  | .403 |
| Item 24 | **-.588**** | .013 | .280 | -.178* | -.108 | .003 | **.086** |  | .525 |
| Turnover intentions (TI) |  |  |  |  |  |  |  |  |  |
| Item 1 |  |  |  |  |  |  |  | .820** | .328 |
| Item 2 |  |  |  |  |  |  |  | .941** | .115 |
| Item 3 |  |  |  |  |  |  |  | .956** | .086 |
| ω | .933 | .584 | .727 | .570 | .665 | .561 | .225 | .933 |  |

*Note.* **p* < .05; ***p* < .01; NF = need fulfillment; λ = Factor loading; δ = Item uniqueness; Target factor loadings are in bold.; ω = model-based omega composite reliability.

**Table S3**

*Parameter Estimates from the Correlates Measurement Model Estimated in Sample 2*

|  | WA (λ) | WS (λ) | δ |
| --- | --- | --- | --- |
| Work addiction (WA) |  |  |  |
| Item 1 | .466** |  | .783 |
| Item 2 | .577** |  | .667 |
| Item 3 | .532** |  | .717 |
| Item 4 | .573** |  | .672 |
| Item 5 | .601** |  | .639 |
| Item 6 | .676** |  | .544 |
| Work satisfaction (WS) |  |  |  |
| Item 1 |  | .903** | .184 |
| Item 2 |  | .631** | .602 |
| Item 3 |  | .866** | .249 |
| Item 4 |  | .608** | .630 |
| Item 5 |  | .808** | .348 |
| ω | .745 | .879 |  |

*Note*. **p* < .05; ***p* < .01; λ = Factor loading; δ = Item uniqueness; ω = model-based omega composite reliability.
